# Green silver nanoparticles curcumin conjugate induced photodynamic therapy of lung cancer and lung cancer stem cells†

**DOI:** 10.1039/d4ra06035k

**Published:** 2025-02-14

**Authors:** Glory Kah, Rahul Chandran, Heidi Abrahamse

**Affiliations:** a Laser Research Centre, Faculty of Health Sciences, University of Johannesburg Johannesburg 2028 South Africa 217004084@student.uj.ac.za rahulc@uj.ac.za habrahamse@uj.ac.za

## Abstract

Lung cancer remains a dreaded disease globally due to its high mortality rates. New cases of lung cancer are estimated at 1.8 million a year, with about 1.6 million deaths. Conventional treatment regimens are inefficient due to their failure to eradicate lung cancer stem cells (LCSCs). LCSCs are noted to self-renew, cause relapse, strengthen metastasis, preserve tumorigenicity, and are very resistant to treatment. This shows the need for a novel treatment modality that can target lung cancer and its stem cells. In this study, a photoactive curcumin–silver nanoparticle–polymer conjugate (Cum–PEG–BpAgNPs) was developed to enhance lung cancer photodynamic therapy (PDT). Lung cancer cells and LCSCs were treated with Cum–PEG–BpAgNPs followed by light irradiation at 470 nm. Post-analytical assays including 3-[4,5-dimethylthiazole-2yl]-2,5-diphenyl tetrazolium bromide, lactate dehydrogenase, adenosine triphosphate, ROS by DCFH-DA, annexin V-FITC/PI cell death studies, and morphological analysis were performed. The characterization analysis confirmed the bio-formulation of Cum–PEG–BpAgNPs conjugate. The LCSCs characterization indicated the presence of LCSCs in the isolated cell population. The biochemical assays post-PDT revealed substantial cytotoxicity when lower concentrations of Cum–PEG–BpAgNPs were used. The IC_50_ value of the conjugate was noted at 4.014 μg mL^−1^ and 2.373 μg mL^−1^ for lung cancer cells and LCSCs, respectively. An elevated ROS production was induced, leading to apoptosis post-PDT. Therefore, Cum–PEG–BpAgNPs could be used in the mediation PDT to eliminate lung cancer cells effectively.

## Abstract 2

Lung cancer remains a dreaded disease globally due to its high mortality rates.

## Graphical abstract

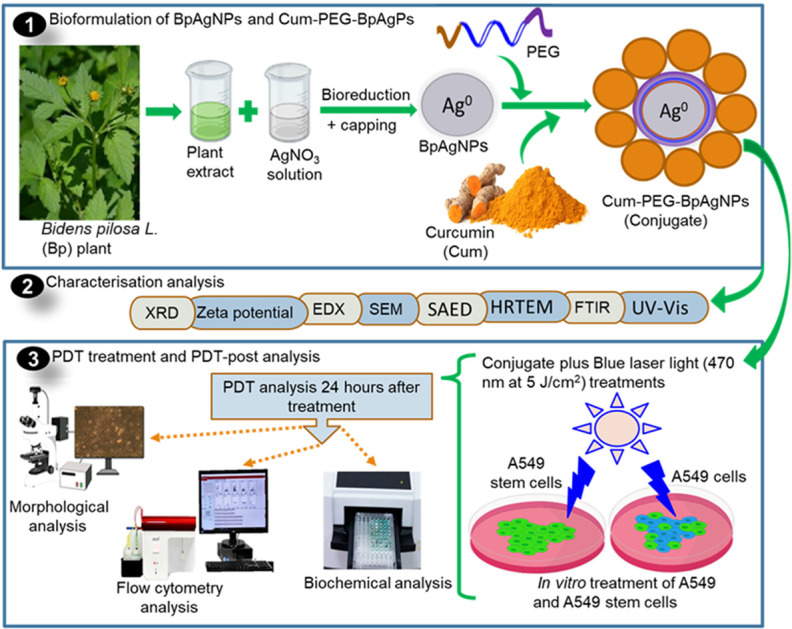

## 1 Introduction

Lung cancer (LC) is any cancer that can develop inside any area in the airways or lungs. The chance of developing LC greatly increases in smokers, accounting for over 70% of cases.^1^ Airborne fine particulate matter (PM2.5) exposure also contributes to lung cancer occurrence in non-smokers. The disease becomes apparent as it advances, though it is rarely noticed at an early stage. Symptoms of lung cancer, such as asthma and cough, hemoptysis, chest infections, unexplained weight loss, and fatigue, are more likely to exacerbate over time.^2^ Two forms of LC exist, mainly non-small cell lung cancer (NSCLC) and small cell lung cancer (SCLC). The NSCLC is of higher prevalence and accounts for over 85% of all cases.^2^ Procedures for lung cancer detection include scans using X-rays that present the tumor simply as a white-grey mass, ultrasound, biopsies, and bronchoscopies that are vital in providing a more conclusive diagnosis and for cancer staging. Nonetheless, present-day treatment for lung cancer consists of radiation, chemotherapy, surgery, and immunotherapy. This treatment is performed as a mixture or a single choice of treatment. Yet, the treatment choice is often determined based on the disease stage and the patient's general wellness.^2^ Despite advancements in lung cancer management, treatment and diagnostic procedures remain tremendously painful, costly, and time-consuming. Though surgery is a greatly effective treatment, long chances of patient survival remain relatively low.^3^ Likewise, most patients do not positively react to treatment, and some develop relapses to the cancer treatments a few months or some years later. The main contributing factor to the relapse in cancer treatment is the failure of cancer therapeutic methods to eradicate cancer stem cells (CSCs) in the cancer tumor tissue. Therapeutic methods like radiotherapy and chemotherapy can stimulate the formation of senescent tumor cells. The up-expressed factors thanks to the senescent cells within the tumor microenvironment might induce signaling pathways that provoke functional and phenotypic alterations of the stem cell (SC) population, hence boosting the SC unchecked growth and plasticity. Again, CSCs are noted to be chemo-resistant, the reason being that they have transporters in their cell membranes that have the ability to release xenobiotic substances into the extracellular space.^4^

LCSCs are specifically linked to particular stem cell biomarkers that are reported to be involved in chemoresistance, metastasis, and preserving tumorigenicity. These biomarkers are valuable targets to be targeted in a more effective lung cancer therapeutic strategy.^4^ Furthermore, treatment like radiotherapy is stipulated for lung cancer cases that have an advanced local inoperable tumor. Yet, radioresistant stem cells present in the tumor contribute to therapeutic failure to radiotherapy, poor prognosis, relapses, and metastasis in patients.^5^ It is affirmed that CD44 and CD133 markers are the major lung cancer stem cell biomarkers.^6,7^ CD44 markers are found to be implicated in signaling cascades that are concerned with tumor progression and initiation and take part in cell migration. The expression of alternate splicing by the CD44 genes is correlated to the expression of different isoforms, which are linked to subtypes of cancer. Thus, CD44 markers are an ideal candidate to be isolated as CSCs surface from carcinoma.^7^ Also, lung cancer CD44^+^ stem cells are able to initiate tumor formation both *in vivo* and *in vitro*. Similarly, CD133^+^ cells are revealed to have a greater ability for tumor initiation, drug resistance, and self-renewal. They also increasingly express Oct4, known to be a usual transcription factor for stem cell embryonic forms.^8^

Photodynamic therapy is viewed as a promising and non-invasive treatment for managing cancer.^9^ PDT makes use of basically three fundamental elements: photosensitizer (PS), oxygen, and a specific wavelength of light that can activate the PS. The activated PS (drug) stimulates an energy transfer cascade to provoke cytotoxicity and hence cell death *via* the generation of reactive oxygen species (ROS) like free radical oxygen and singlet oxygen.^10,11^ Compared to traditional cancer treatment like chemotherapy, PDT has more spatial selectivity and invasiveness as ROS is only induced in the areas that were exposed to light irradiation.^12,13^ This attribute makes PDT present quite low systemic toxicity. Likewise, PSs employed in PDT are noted to have limited harmful ability when not exposed to light and will bring about less systemic damage if build-up within nonspecific tissues. In addition, the PS-activating light source applied in PDT is nonionizing compared to that used in radiotherapy. This makes PDT light not pose a hazardous risk to tissue in the absence of the PS drug. Also, surgical tumor resection is noted to be correlated with elevated recurrence. Consequently, PDT could securely be repeated whenever necessary without the risk of incurring damaging effects on normal tissues.^9,13^

Notwithstanding the aforementioned benefits of PDT, low therapeutic outputs are also still linked to PDT. The main chief factor is that the PS may not actively be deposited on the tumor-specific side following *in vivo* administration, similar to chemotherapeutic drugs exploited in traditional cancer therapy.^10,14^ Similarly, significant non-specific absorption of PS by other tissues drastically reduces the therapeutic effects of PDT.^13^ Besides, most PSs have poor solubility, rapidly deplete upon photoactivation, sometimes present momentary retention within the target tissues, and have a restricted excitation window. However, nanotechnology provides distinct advantages compared to small molecular drugs in that nanomaterials can facilitate the delivery of great amounts of payload PSs into the targeted tissue, offer excitation wavelength flexibility, improve the duration of drug retention in tissue of interest, capable of producing regenerative ROS, and offer an enhanced effect on the immune system.^15^ This therefore shows that the improvement of PDT *via* nanotechnology techniques could logically help in overcoming the drawbacks of PDT. Nanotechnology techniques that focus on improving the efficacy of PS, such as chemical conjugation methods, have been suggested by researchers. This technique can chemically modify the PS attributes, including solubility, improve the efficacy of generating ROS, and help to block the PS from self-quenching. This may suggest that PDT could be desirable for malignant tumors where repeated therapy is needed. Therefore, PDT could be able to treat most tumors that are resistant to traditional treatments.^13^

A natural polyphenol called curcumin has significantly drawn the attention of scientists, particularly because of its distinct therapeutic characteristics, including anti-inflammatory, antifungal, antioxidant, antibacterial, and anti-carcinogenic effects, and it can be used as PS in cancer PDT.^16^ However, the poor solubility of curcumin tremendously hinders its *in vivo* therapeutic potential. This results in inadequate bio-absorption, fast body clearance, and limited intrinsic activity. In this regard, nanotechnology methods where curcumin can be conjugated to form a nanoparticle–polymer composite have been encouraged. This method is simple and efficient and can improve the solubility and bioavailability of curcumin without lessening its medical qualities.^17^ Nanomaterials, especially silver nanoparticles (AgNPs), have garnered much interest recently for their diverse applications in therapy owing to their distinct biological, chemical, physical, and optical qualities.^18^ Likewise, metallic nanoparticles like AgNPs, when conjugated with PSs, can function as drug carriers, facilitating the delivery of the drug to the intended targeted side. The targeted delivery of the conjugated drug to organs like the lung can lengthen its duration of treatment, enable the regulation of the treatment dose, lower the likelihood of patients encountering harmful toxicity effects, and reduce the chances of complications. Besides, the targeted delivery of the drug may also guarantee that it reaches the affected area, thereby reducing the likelihood of its distribution to unintended tissue and organs.^19^ Moreover, the therapeutic application of AgNPs which is obtained *via* the green synthesis method is encouraged since the method makes use of eco-friendly materials, including fungi, bacteria, and plants, for nanoparticle formulations. This green method is distinctively environmentally safe, less expensive, and generates no toxicity that can cause environmental and biological risks compared to the chemical and physical reduction procedure.^18,20^ Besides, the AgNP synthesis method that explores plants benefits from the advantages that bioactive compounds from the plant extracts such as vitamins, amino acids, proteins, and enzymes, may dually work reduction and capping agents during synthesis.^20^ This study thus explores the *Bidens pilosa* (Bp) plant in the green synthesis of AgNPs.

The Bp plant, often called blackjack is a perennial plant that is commonly found in fields as a weed. However, the plant is palatable, edible, safe, and grows easily, thus promoting its cultivation for food (vegetables, tea, and sauce) and medical purposes in some countries in Africa, including Zimbabwe, Nigeria, Benin, Kenya, Mozambique, Cameroon, Botswana, Zambia, the Congo, and South Africa.^21–23^ Also, Bp is known for its medicinal ability to heal a variety of illnesses, including respiratory infections, diarrhea, dysentery, wounds, and indigestion.^24^ Bp can lower the incidence of colon cancer, anemia, blindness, immune-related illnesses, and cardiovascular disease due to its rich content of phytochemicals.^23^ Bp extract also exhibits pharmacological attributes such as antifungal, antiulcer, and antibacterial effects.^25^ The bioactive chemical compound paclitaxel, isolated from Bp leaf extract is noted to have anticancer properties.^26^ Other bioactive compounds in the group of major compounds, such as flavone glycosides, okanin, pheophytins, chalcones, polyacetylene glycosides, phytosterols, fatty acids, terpenes, phenolic acids, glycosides, aurones, flavonoids, and polyacetylenes, have also been isolated from the Bp plant.^25,27^ These compounds could play a role in the green synthesis of AgNPs by acting as reducing and capping reagents. This implies that AgNPs obtained using Bp plant can be cost-efficient, eco-safe, and more suitable for therapeutic application.

We, therefore, report here a method where AgNPs were obtained using Bp aqueous extract and then conjugated with curcumin (Cum). The curcumin–silver nanoparticle–polymer conjugate (Cum–PEG–BpAgNPs) formulated were subjected to *in vitro* therapeutic application against lung cancer and their stem cells. The Cum–PEG–BpAgNPs may offer a better photodynamic potential for eradicating lung cancer and its stem cells. This is particularly important since traditional lung cancer treatment offers severe after-effects and does not seem to annihilate LCSCs responsible for lung cancer initiation.

## 2 Materials and methods

### 2.1 Kits and reagents

CD44-FITC (order no. 130-113-334, Miltenyi Biotec), CD133/2 antibody, anti-human, PE (130-113-748), CD133 MicroBead Kit, human-lyophilized (130-097-049), and CD44 MicoBeads Kit were procured from Miltenyi Biotec, Bergisch Gladbach, Germany. Curcumin (C1386-50G), ultrathin lacey carbon-supported copper grid (TEM-LC300CUUL), nitric acid silver (AgNO_3_ ≥ 99.9999%) metals basis (204390-50G), qualitative filter papers (WHA1001125), 2′,7′-dichlorofluorescein diacetate (D6883-50MG), epidermal growth factor (SRP3027-500UG), basic fibroblast growth factor (GF003AF-100UG), and thiol-polyethylene glycol-amine (HS-PEG2K-NH_2_, JKA5143) were obtained from Sigma-Aldrich and Hoechst 33342 (2′-[4-ethoxyphenyl]-5-[4-methyl-1-piperazinyl]-2,5′-bi-1*H*-benzimidazole trihydrochloride trihydrate) from Thermo Scientific.

### 2.2 Plant collection and preparation

*Bidens pilosa* (Bp) plant was collected on March 13, 2024, from Randburg, South Africa. Dr Ashton Welcome from the Department of Botany at the Auckland Park Kingsway Campus, University of Johannesburg, South Africa determined its authenticity. It is now housed at the University of Johannesburg Herbarium (JRAU) with reference KAH1 2024. The plants' leaves were collected, rinsed in distilled water (dH_2_O), and then chopped into fine pieces for drying. The dried leaves were transformed into powder with the help of a grinder. The powdered plant sample was weighed (20 g) and boiled in 150 mL of dH_2_O for 1 hour (h) under magnetic stirring. Subsequently, plant filtrate was collected from the plant mixture by filtration using a filter paper (Sigma, WHA1001125, Johannesburg, South Africa).

### 2.3 BpAgNPs biosynthesis

Nitric acid silver (AgNO_3_ ≥ 99.9999% metals basis, Sigma, 204390, Johannesburg, South Africa) was prepared in a 90 mL dH_2_O to 5 mM solution in a Schott bottle (250 mL). The Bp water extract (10 mL) was added gently to the AgNO_3_ solution. The mixture was kept under constant magnetic stirring for 3 days at 40 °C for the formulation of *Bidens pilosa* silver nanoparticles (BpAgNPs) to complete. Afterward, the obtained reaction mixture was aliquot in 50 mL tubes and subjected to twice repeated centrifugation for 40 minutes (mins), at 4900 rpm and 25 °C. The pellet obtained was then re-diluted with sterile dH_2_O and washed thrice by centrifugation. The BpAgNPs pellet was frozen over 24 h at −80 °C and freezer-dried for subsequent analysis.

#### 2.3.1 Conjugation of BpAgNPs with curcumin

BpAgNPs at a concentration of 20 mg mL^−1^ in dH_2_O were mixed with 10 mg of thiol-polyethylene glycol-amine (PEG) (HS-PEG2K-NH_2_, Sigma, JKA5143, Johannesburg, South Africa) and sonicated for 1 h, then allowed to stand undistributed at room temperature for 24 h to permit the surface coating and ligand exchange between BpAgNPs with PEG. After that, 20 mg mL^−1^ of curcumin dissolved in 1× phosphate buffer saline (1× PBS) containing 0.05 N of sodium hydroxide was added to the PEGylated BpAgNPs (PEG–BpAgNPs) solution, and the mixture was then subjected to 20 min of sonication without heat. Subsequently, the solution was kept for 24 h in the dark at 4 °C to enable curcumin to bind with PEG–BpAgNPs. Three serials of centrifugation followed this at 18 000 rpm for 45 min. The pellet obtained was diluted with sterile dH_2_O and also centrifuged thrice. The supernatant with any unbounded or free molecules was completely removed, and the pellet containing the conjugate (Cum–PEG–BpAgNPs) was kept within a week at 4 °C for characterization analysis.

### 2.4 Characterization analysis

Characterization of nanomaterials is the keystone for validating the development and formation of adoptable, safe materials for different applications.^28^ We thus performed the following techniques to validate the chemical and physical attributes of the bio-synthesized BpAgNPs and their curcumin conjugate.

#### 2.4.1 UV-vis spectroscopy

The distinct optical attributes of Bp extract, BpAgNPs, PEG–BpAgNPs, PEG, and Cum–PEG–BpAgNPs were verified. Each sample for analysis was diluted with dH_2_O and homogenized. Then 1.5 mL of each homogenized preparation was transferred to a UV-grade cuvette and analyzed by exploring the UV-vis spectrophotometer instrument (Genova Nano, Jenway Spectrophotometer, Lasec, Cape Town, South Africa) at wavelength values ranging from 200 to 800 nm.

#### 2.4.2 Fourier-transform infrared (FTIR) spectroscopy

The main active chemical groups that could be involved in BpAgNPs, PEG–BpAgNPs, and Cum–PEG–BpAgNPs formation were established *via* FTIR. Freeze-dried samples were mixed with potassium bromide (KBr) in a 1 : 100 ratio, ground into a homogenous powder, and pelleted. Each pellet containing the sample was examined *via* the FTIR instrument (PerkinElmer Spectrum 100 FTIR spectrometer, USA) at wavelengths spanning from 400 to 4000 cm^−1^.

#### 2.4.3 Energy dispersive X-ray (EDX) and scanning electron microscopy (SEM) analysis

SEM and EDX analysis were achieved using powdered films of BpAgNPs, PEG–BpAgNPs, and Cum–PEG–BpAgNPs samples. These samples were spread uniformly on carbon tape stuck to SEM sample holders. A sputter coater (Quorum Q300T ES, East Sussex, UK) was then utilized to coat the samples with carbon. The samples were imaged with the SEM machine (TESCAN Vega 3, Brno, Czech Republic), and EDX analysis was performed with the micro-X-ray instrument (X-MAX EDX Oxford, Oxford, UK) coupled with the TESCAN system.

#### 2.4.4 High-resolution transmission electron microscopy and selected area electron diffraction (HRTEM/SEAD)

The samples for HRTEM and SEAD were sonicated in dH_2_O, and drops of the sonicated material were deposited to air dry on TEM grids. Each grid was staged on an HRTEM sample holder and examined *via* an electron microscope (JEOL JEM-2100, 80–200 kV, JEOL Ltd, Japan, with EELS). Micrographs were taken, and the SEAD configuration produced by the sample was taken. The SEAD configuration and the micrographs were examined by exploring the ImageJ 1.51p software to affirm the crystallinity and the size of the material under investigation.

#### 2.4.5 X-ray diffractometry (XRD)

Lyophilized BpAgNPs, PEG–BpAgNPs, and Cum–PEG–BpAgNPs samples were finely powdered in a mortar. The powdered samples were placed onto XRD sample plates and analyzed using the XRD machine (Malvern Panalytical, Aldwych, London) that was set to operate at an angular range of 4 to 90°, 2*θ* angle, and radiation of 1.5443 Å Cu Kα.

#### 2.4.6 Zeta potential (*ζ*-potential) analysis

An electric potential produced on a particle's surface is known as the *ζ*-potential. It can be measured by determining the velocity of charged species in samples under analysis as they attempt to move through an electric field. Important details about the net surface charge (state) and relative stability of nanomaterials can be obtained from zeta potential.^29^ BpAgNPs, PEG–BpAgNPs, and Cum–PEG–BpAgNPs samples were diluted in dH_2_O and subjected to 10 min of sonication. The suspension was pipetted into a zeta dip cell and analyzed with the zeta machine (Malvern Zetasizer 2000, Malvern, UK) kept at 25 °C and supplied with a laser beam from a steady source.

### 2.5 Cell line and maintenance

Cell lines (A549, ATCC® CCL-185) were obtained and seeded in complete Roswell Park Memorial Institute 1640 media (RPMI-1640, R-8758, Sigma, South Africa) with 0.5% amphotericin B, penicillin/streptomycin, respectively (A2942, P4333, Sigma, South Africa), and 10% foetal bovine serum and kept in an incubator (with 85% humidity, 37 °C, and 5% CO_2_) to grow. After that, macro- and microscopic examinations were performed every 24 h. Media change was done depending on the change in media color and cell density. Grown and adherent cells were harvested and preserved at −80 °C in cryo-medium or sub-cultured for further analysis. In addition, the A549 stem cell side pollution was grown in complete media [Dulbecco's Modified Eagle Medium/Nutrient Mixture F-12 (DMEM/F-12), N6658, Sigma, South Africa] containing 0.5% of amphotericin B, 0.5% of penicillin/streptomycin, and growth factors [10 ng mL^−1^ of epidermal growth factor (SRP3027, Sigma, South Africa)] and basic fibroblast growth factor (GF003AF, Sigma, South Africa), respectively. It should be noted that the A549 cells were obtained at passage 25 and were grown to passage 32 for experimental analysis. The passage number of A549 stem cells was maintained up to passage 6 for experimental analysis. This was to ensure that the A549 stem cells were not differentiated into A549 cells.

### 2.6 CD44^+^ and CD133^+^ A549 SC sorting

An immunomagnetic bead sorting procedure was exploited to isolate stem cells (CD133^+^ and CD44^+^ cells) from the A549 cell lines population. The Miltenyi Biotec Magnetic Activated Sorting System (MACS) MicroBead kits, numbering CD133 (130-097-049) and CD44 (130-095-194), were used. A549 confluent cells from T175 flasks were dissociated using TrypLE™ (12563-029, Gibco, South Africa), harvested, and washed by centrifugation at 3000 rpm for 10 min in HBSS (Hank's Balanced Salt Solution, 55021C, Sigma, South Africa). The cells were counted and diluted in 1× PBS isolation stem cell buffer composed of 0.5% bovine serum albumin (BSA, A2153, Sigma, South Africa) and 2 mM ethylenediaminetetraacetic acid (EDTA). Subsequently, the cells were treated with the FcR blocking reagent, and microbeads were added as directed by the manufacturer. The tube with the cells was incubated in the dark at 8 °C for 15 min while being gently mixed by being maintained constantly to rotate on a MACSmix Rotator. Incubating the cells in FcR blocking reagent helps to improve the purity of target cells by increasing the specificity of antibody or microbead labeling. The isolation buffer was then added to the cells and centrifuged at 300 rpm for 10 min. The supernatant was removed, and cells were re-suspended in the isolation buffer (10^7^ cells: 500 μL of buffer). The separation MACS column fitted to a magnetic MACS separator was then utilized to separate the stem cells (CD133^+^ and CD44^+^ cells) from the cell population. This is based on the principle that unlabeled cells are directly eluded out of the column, and the labeled cells (CD133^+^ and CD44^+^ cells) attached to the magnetic microbeads are retained in the column thanks to the magnetic attraction of the magnetic separator. The column was taken out of the magnetic separator, and fitted into a proper collection tube, and the trapped cells were flushed out of the column using a plunger. The CD133^+^ and CD44^+^ cells collected were seeded in complete DMEM/F-12 media.

### 2.7 Immunofluorescence of A549 stem cells (A549 SC) surface markers

The CD133^+^ and CD44^+^ side populations at 3 × 10^5^ cells per 2 mL of complete media were plated on glass coverslips in 3.5 cm culture plates allowed in the incubator (with 85% humidity, 37 °C, and 5% CO_2_) to proliferate and attached. The attached and confluent cells were washed with 1× PBS three times, followed by fixation in 4% paraformaldehyde for 15 min. The cells were then washed in 1× PBS and allowed to sit for 45 min in 1 mL of blocking buffer (0.5% BSA in 1× PBS). The cells were maintained for 45 min in this blocking buffer to help reduce the non-specific binding of the conjugated antibodies while also helping to promote the sensitivity and specificity of the staining procedure. Thereafter, the cells were stained using the CD44-FITC antibody conjugate (cat. # 130-113-334, Miltenyi Biotec, USA) and CD133-PE antibody conjugate (cat. # 130-113-186, Miltenyi Biotec, USA) prepared in 1× PBS in a volume ratio of 1 : 50 parts and allowed for 1 h at 8 °C in the dark. Unbound antibodies were rinsed off from the cells by washing the cells in three repeats using 1× PBS. The cells were stained in 1 μg mL^−1^ of 4′,6-diamidino-2-phenylindole (DAPI) (cat. # D1306, Invitrogen, South Africa) for 10 min, washed in 1× PBS, and rinsed with sterile dH_2_O. The coverslips with stained cells were then sealed on a mounting medium on glass slides and observed using the Olympus BX41 (Tokyo, Japan) fluorescent microscope. Images were captured with the assistance of the Olympus XC10 digital camera that is linked with the CellSens Olympus imaging program.

### 2.8 Hoechst 33342 assay

The A549 SC side populations in complete media were grown in 3.5 cm plates on coverslips to be attached over 24 h. The media was then aspirated, and the cells were washed thrice with 1× PBS. The Hoechst (14533, Sigma, South Africa) reagent (200 μL), prepared at a working concentration of 3 μg mL^−1^ in pre-warmed serum-free media was added to the cells and incubated for 45 min. Afterward, the cells were washed with 1× PBS three times, and Hoechst-treated cell slides were mounted on glass slides with mounting medium and sealed at the edges using transparent nail polish. A microscopic investigation of the slides was performed using the DAPI filter.

### 2.9 Cellular uptake analysis

The cellular uptake analysis (localization analysis) of the drug (Cum–PEG–BpAgNPs) by the A549 and A549 SCs was validated *via* a fluorescence microscopic procedure. The A549 and A549 SCs were seeded on sterile glass coverslips in 3.5 cm small culture plates at 5 × 10^5^ in 2 mL of culture media and maintained in the incubator for 24 h to grow and attach to the coverslips. The culture medium was discarded, and the cells were rinsed with 1× PBS. Then, the cells were treated in 2 mL of pre-warmed media containing 15 μg mL^−1^ of Cum–PEG–BpAgNPs in an incubator for 4 h at 85% humidity, 5% CO_2_, and 37 °C. The cells were washed in 1× PBS thrice to remove any trace of non-absorbed Cum–PEG–BpAgNPs. The cells were fixed for 15 min at room temperature in 1 mL of paraformaldehyde (4%) and then washed with PBS. The cells were permeabilized for 15 min in 1 mL Triton X-100 (0.5%) in 1× PBS, washed, and stained at 4 °C in 150 μL of each pre-warmed probe [65 nM endoplasmic reticulum tracker (ER Tracker, 8787S, Cell Signaling Technology), 65 nM lysosome tracker (Lyso Tracker, L7526, Invitrogen), and 100 nM mitochondria tracker (Mito Tracker M7514, Invitrogen)] for 30 min. The cells were washed, counter-stained at room temperature for 10 min in 1 μg mL^−1^ of DAPI (200 μL), and again washed thrice in 1× PBS. The coverslips with stained cells were then inverted onto microscopic slides containing a drop-mounting medium. The edges of the coverslips were rapidly sealed with clear nail polish to avoid drying out samples. The stained cells were observed with the 63× objective of the Zeiss microscope (Carl Zeiss Microscopy GmbH, Jena, Germany) with the help of specific filters with different excitation (Ex) and emission (Em) wavelengths: Ex341/Em452 nm for DAPI, Ex467/Em571 nm for Cum–PEG–BpAgNPs, Ex504/Em511 nm for the ER Tracker, Ex380/Em576 nm for the Lyso Tracker, and Ex490/Em516 nm for the Mito Tracker.

### 2.10 Laser irradiation parameters

We made use of a 470 nm blue light laser from the national laser center (CSIR, NLC, Pretoria, South Africa), which was powered with a constant power supply (input: 85–264 VAC, 47–63 Hz, 2 A). The laser treatment was performed for 1 min 2 seconds at 5 J cm^−2^, 80 mW cm^−2^, and in the dark, free of any source of light.

### 2.11 Dark cytotoxicity, PDT, and IC_50_ treatment groups

The drug (Cum–PEG–BpAgNPs) utilized in this study was subjected to dark cytotoxic analysis to determine the drug concentration that would cause the least amount of toxicity or none at all. The determined concentrations were then exploited in A549 and A549 SC PDT treatment. The IC_50_ concentration following PDT was also determined and utilized in A549 and A549 SC ROS and annexin V-FITC/PI cell death studies. All the analyses were performed in triplicate according to the experimental groups following the dark cytotoxic, PDT, and IC_50_ treatments as indicated in Table 1.

**Table 1:** Dark cytotoxicity, PDT and IC_50_ treatment groups^a^

Experimental groups (*n* = 3)	Cells and treatment description		
	Cum–PEG–BpAgNPs (μg mL^−1^) dark toxicity	Cum–PEG–BpAgNPs (μg mL^−1^) + PDT	IC_50_ treatment
1	Cells only	Cells only	Cells only
2	Cells + 0.75	Cells + laser (L)	Cells + L
3	Cells + 1.5	Cells + 0.75 + L	Cells + IC_50_ treatment
4	Cells + 3.12	Cells + 1.5 + L	
5	Cells + 6.25	Cells + 3.12 + L	
6	Cells + 12.5	Cells + 6.25 + L	
7	Cells + 25	Cells + 12.5 + L	
8	Cells + 50		
Total sample size	24	21	9

a Analysis in each experimental group all performed in triplicate (*n* = 3), cells (A549 and A549 SC), and laser light irradiation (L).

### 2.12 Dark cytotoxicity/laser irradiation dose–response biochemical assays

The Cum–PEG–BpAgNPs (drug) utilized in both the dark cytotoxicity and PDT treatments was kept at 4 °C and utilized within a week following its synthesis. The Cum–PEG–BpAgNPs at doses 0, 0.75, 1.5, 3.12, 6.25, 12.5, 25, and 50 μg mL^−1^ were investigated for their dark cytotoxicity ability on A549 and A549 SC (co-side population of CD44^+^ and CD133^+^). A549 and A549 SC at 1 × 10^4^/100 μL of growth media were seeded to attach. They were washed in HBSS and treated with the above-mentioned concentrations of Cum–PEG–BpAgNPs for 24 h. Thereafter, the cells were washed thrice to remove the non-absorbed drug, 100 μL of media was added to the cells, and they were incubated for another 24 h followed by MTT, LDH, and ATP analysis.

For the PDT procedure, A549 and A549 SC were seeded at 1 × 10^4^/100 μL in 96-well plates to attach overnight. The cells were washed with HBSS, treated with complete media containing minimal or non-toxic concentrations of Cum–PEG–BpAgNPs (0.75, 1.5, 3.12, 6.25, and 12.5 μg mL^−1^) for 4 h. The cells were washed thrice with HBSS to get rid of any unabsorbed drug by the cell and then irradiated. The cells were again washed thrice with HBSS and incubated in 100 μL of complete media for 24 h, followed by MTT, LDH, and ATP analysis.

The MTT assay was achieved by treating the cells at post-treatment with 10 μL (0.5 mg mL^−1^) MTT reagent (3-[4,5-dimethylthiazole-2yl]-2,5-diphenyl tetrazolium bromide assay, 11465007001 Roche, Sigma, South Africa) contained in 90 μL of serum-free media for 4 h. The MTT solubilization buffer (100 μL) was added to the mixture, and the treated cell mixture was kept for 24 h in the incubator before being analyzed with the plate reader at 550 nm. The lactate dehydrogenase (LDH) assay (Non-radioactive CytoTox 96, PRG1780, Promega, USA) was done by adding 50 μL of LDH test reagent to the 50 μL of suspension media obtained from the cells 24 h post-treatment. This mixture was then kept in the dark at room temperature for 30 min followed by analysis at 490 nm using the microplate reader. Also, the adenosine triphosphate (ATP) test was performed by adding 50 μL of ATP reagent (ATP CellTiter-Glo® reagent, G968A, Promega, USA) to an equal volume of cell suspension at 24 h post-treatment. The mixture was then agitated gently to induce lysis for 4 min, incubated in the absence of light at room temperature, and the samples were analyzed by the plate reader (PerkinElmer plate reader, VICTOR Nivo™, Johannesburg, South Africa).

### 2.13 Morphological studies at post-treatment

A549 and A549 SCs were seeded at 5 × 10^5^ cells in a 3.5 cm culture dish to attach. After 24 h the cells were washed with HBSS and treated with Cum–PEG–BpAgNPs for 4 h followed by irradiation treatment. The cells were incubated for 24 h in complete media and then screened *via* an inverted microscopic (Wirsam, Olympus CKX41, Tokyo, Japan) for any morphological alternations of the cells.

### 2.14 Reactive oxygen (ROS) analysis

ROS detection was performed quantitatively using the 2′,7-dichloro-dihydrofluorescein diacetate (DCFH-DA, D6883, Sigma, South Africa) assay. A549 and A549 SC were seeded in 96 well plate at 1 × 10^4^ cells/well/100 μL of media and incubated to attach over 24 h. The cells were washed with HBSS and treated for 45 min in 100 μL serum-free media containing 20 μM DCFH-DA reagent. Thereafter, the cells were washed with HBSS and treated with Cum–PEG–BpAgNPs IC_50_ concentration for 4 h followed by irradiation treatment. After 1 h post-treatment, the activated ROS fluorescence signal was validated using the plate reader at Ex485/Em535 nm.

### 2.15 Cell death mechanism

The mechanism of cell death was determined post-treatment PDT using the apoptosis–necrosis kit (annexin V-FITC and propidium iodide (PI), 559763, BD Pharmingen™, USA). The cells were cultured at 5 × 10^5^ cells/2 mL of media in 3.5 cm sterile plates. The attached and confluent cells were treated with the drug IC_50_ for 4 h and irradiated. Twenty-four h post-treatment, the cells were washed in 1× PBS, unattached using TrypLE™, and washed again in 1× PBS. The cells were homogenized in 1× binding buffer at 1 × 10^6^ cells per mL, and 100 μL of the cell suspension was transferred to flow cytometry tubes and then stained with 5 μL of annexin V-FTIC and PI reagent in the dark at room temperature for 10 min. The tubes were top-up with 400 μL of binding buffer, gently mixed, and analyzed with the flow cytometer (BD Accuri™ C6 Plus, Erembodegem, Belgium).

### 2.16 Data processing

The data obtained in this study was processed *via* OriginPro 2018 and the Prism 7.0 GraphPad software program. The statistical significance level was set at *p* < 0.05 and the significance difference between the control and experiment groups was validated by Dunnett's one-way ANOVA (Analysis of Variance) method. Dose-dependent significance above or below the control value was determined based on each protocol.

## 3 Results

### 3.1 UV-vis spectroscopy

**Fig. 1:**
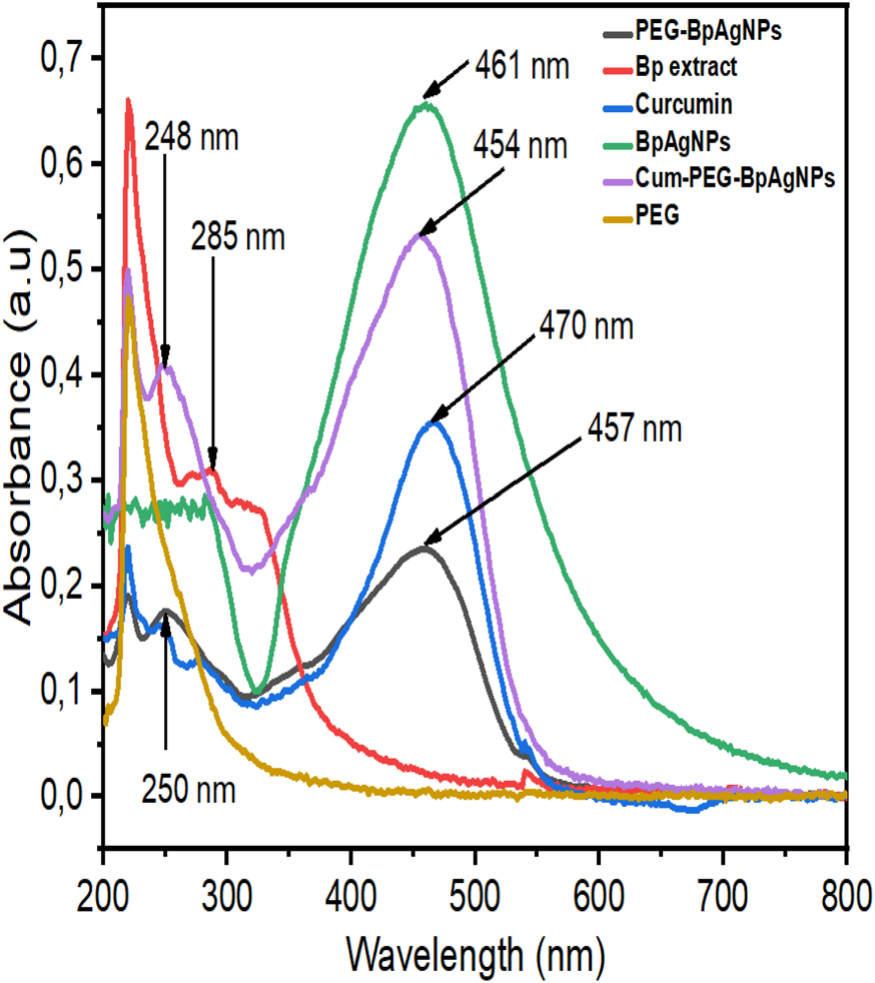
UV-vis absorption spectra for the Bp aqueous extract, BpAgNPs, PEG, PEG–BpAgNPs, curcumin, and Cum–PEG–BpAgNPs.

Fig. 1 shows the UV-vis spectroscopy absorption spectra of the samples. It is confirming the formation of BpAgNPs, PEG–BpAgNPs, and Cum–PEG–BpAgNPs. Prominent peaks for PEG–BpAgNPs were noted at 250 and 457 nm. The Bp aqueous plant extract showed a peak at 285 nm, and curcumin had a noticeable peak at 470 nm. Moreover, the bio-formulated silver nanoparticles (BpAgNPs) had a prominent peak at 461 nm, while the conjugate (Cum–PEG–BpAgNPs) had peaks radiating at 248 and 454 nm.

### 3.2 FTIR spectroscopy

**Fig. 2:**
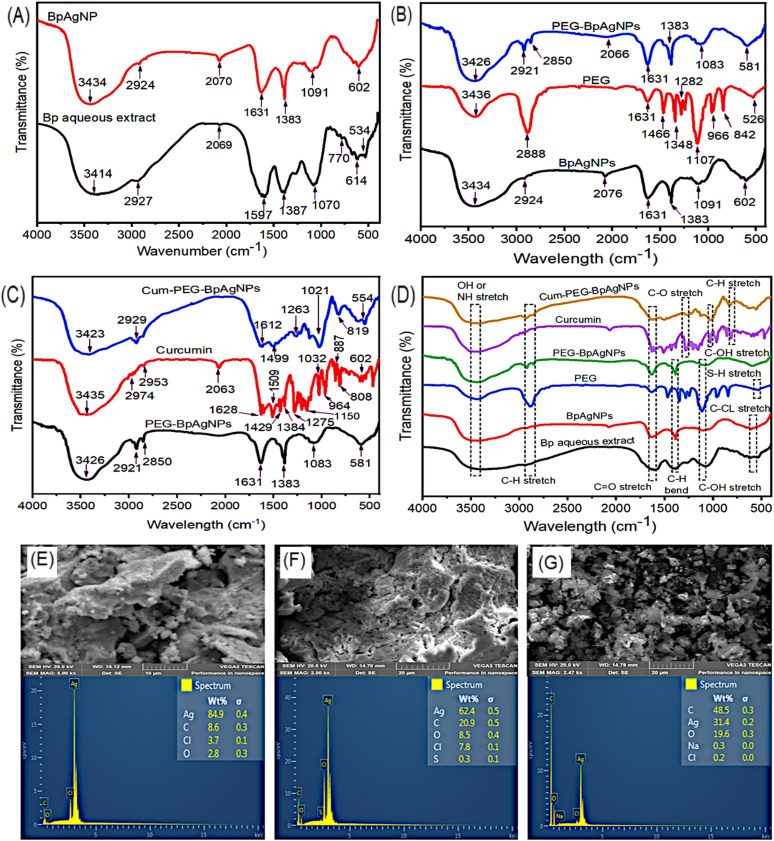
The FTIR spectra, SEM micrograph, and EXD of analyzed samples: (A) spectra for the synthesis of BpAgNPs from Bp plant aqueous extract, (B) spectra of formation of PEG–BpAgNPs from BpAgNPs and PEG, (C) spectra of formation of Cum–PEG–BpAgNPs from PEG–BpAgNPs and curcumin, (D) spectra indicating major functional group in BpAgNPs, PEG–BpAgNPs, and Cum–PEG–BpAgNPs formation. (E) SEM and EDX of BpAgNPs, (F) SEM and EDX PEG–BpAgNPs, and (G) SEM and EDX Cum–PEG–BpAgNPs.

Fig. 2 shows the obtained FTIR spectra confirming the functional groups that were involved in the formulation of BpAgNPs, PEG–BpAgNPs, and Cum–PEG–BpAgNPs. The intense signals of major functional groups were observed at around 3436, 3435, 3434, 3426, 3423, and 3414 cm^−1^ (OH or NH stretching vibration); 2974, 2953, 2927, 2927, 2924, 2921, 2988, and 2850 cm^−1^ (C–H stretching vibrations); 1631, 1628, 1612, and 1597 cm^−1^ (C=O stretching); 1387, 1384, and 1383 cm^−1^ (C–H bending vibrations); 1282, 1275, and 1263 cm^−1^ (C–O stretching); 1107, 1091, 1083, 1070, and 1019 cm^−1^ (C–OH stretching); 813 cm^−1^ (C–H stretch); 614 cm^−1^ (C–Cl); and 526 cm^−1^ (S–H vibration) (Fig. 2D).

The Bp aqueous extract (Fig. 2A), has functional groups with peak intense at 3414 cm^−1^ (O–H or N–H stretching vibration), 2927 cm^−1^ (C–H stretching vibration), 1597 cm^−1^ (C=O or C=C stretching), 1387 cm^−1^ (C–H bend vibration), 1070 cm^−1^ (C–OH stretching), 770 cm^−1^ (C=C bending), 614 and 534 cm^−1^ (C–Cl vibration). The peak for the bio-formulated BpAgNPs was obtainable at 3434 cm^−1^ (O–H or N–H stretching vibration), 2924 cm^−1^ (C–H stretching vibration), 1631 cm^−1^ (C=O or C=C stretching), 1383 cm^−1^ (C–H bend vibration), 1091 cm^−1^ (C–OH stretching), and 602 cm^−1^ (C–Cl stretching) (Fig. 2A). In Fig. 2B, major peaks for functional groups of interest were noted at 3436 cm^−1^ (N–H stretching vibration), 2888 cm^−1^ (C–H stretching vibration), 1631 cm^−1^ (C=O stretching), 1348 cm^−1^ (C–H bend vibration), 1282 cm^−1^ (C–O stretching vibration), 1107 cm^−1^ (C–OH stretching vibration or C–O–C stretching vibration), 842 cm^−1^ (C–S vibration), and 526 cm^−1^ (H–S vibration) for PEG. Also, the FTIR major peaks for the PEGylated nanoparticles (PEG–BpAgNPs) (Fig. 2B) were confirmed at 3426 cm^−1^ (N–H stretching vibration), 2921 cm^−1^ (C–H stretching vibration), 1631 cm^−1^ (C=O stretching), 1383 cm^−1^ (C–H bend vibration), 1083 cm^−1^ (C–OH stretching vibration), and 581 cm^−1^ (H–S vibration).

In addition, Fig. 2C shows major peaks for curcumin recorded at 3435 cm^−1^ (O–H stretching vibration), 2974 and 2953 cm^−1^ (–CH_3_ and –CH_2_ vibrations), 1628 cm^−1^ (C=O stretching vibration), 1509 cm^−1^ (C=C vibration), 1429 cm^−1^ (C–H bend vibration), 1384 cm^−1^ (CH_3_ bending), 1275 and 1150 cm^−1^ (C–O stretching vibration), 1032 and 964 cm^−1^ (C–OH stretching vibration), 887 (C–O vibration) and 808 cm^−1^ (C–H vibrations). The FTIR intense peaks for the formulated Cum conjugate (Cum–PEG–BpAgNPs) (Fig. 2C) were observed at 3423 cm^−1^ (O–H stretching vibration), 2929 cm^−1^ (C–H stretching vibration), 1612 cm^−1^ (C=O stretching vibration), 1499 cm^−1^ (C–H bend vibration), 1263 cm^−1^(C–O stretching vibration), 1021 cm^−1^ (C–OH stretching vibration), 819 cm^−1^ (C–S vibration), and 554 cm^−1^ (S–Ag vibration). Hence, Fig. 2D shows the major functional groups involved in the formation of BpAgNPs, PEG–BpAgNPs, and Cum–PEG–BpAgNPs.

### 3.3 SEM and EDX

**Fig. 3:**
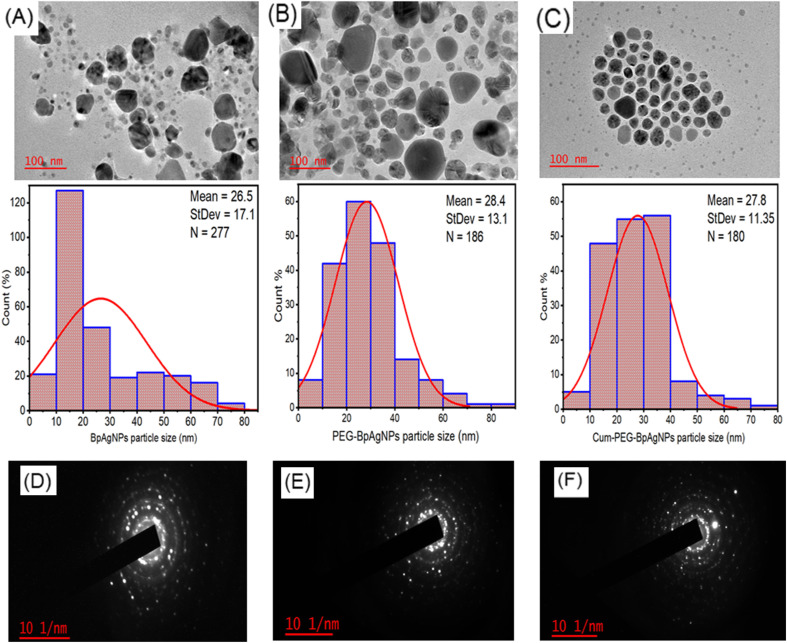
HRTEM micrograph, particle size histogram, and SAED patterns for BpAgNPs, PEG–BpAgNPs, and Cum–PEG–BpAgNPs: (A) morphology and particle histogram of BpAgNPs, (B) morphology and particle histogram PEG–BpAgNPs, (C) morphology and particle histogram Cum–PEG–BpAgNPs, (D) SEAD pattern of BpAgNPs, (E) SEAD of PEG–BpAgNPs; (F) SEAD of Cum–PEG–BpAgNPs.

The SEM micrographs for the formulated BpAgNPs, PEG–BpAgNPs, and Cum–PEG–BpAgNPs showed clouds of polydispersed materials (Fig. 2E–G), respectively. The micrograph shows clusters or aggregated nanoparticles, and the factor attributed to this can be the SEM sample preparation technique. However, the particle sizes were determined *via* the HRTEM, which produced more precise and well-resolved images (Fig. 3). The EDX disclosed that the percentage weight of Ag in the BpAgNPs was 84.9% and a small amount of carbon (8.6%), chlorine (3.7%), and oxygen (2.8%) (Fig. 2E). The PEG–BpAgNPs (Fig. 2F) had 62.4% Ag, 20.9% carbon, 8.5% oxygen, 7.8% chlorine, and 0.3% sulphur. The EDX for Cum–PEG–BpAgNPs (Fig. 2G) had the lowest percentage of Ag (31.4%) and the highest proportion of carbon and oxygen at 48.5% and 19.6%, respectively; a small proportion of sodium and chlorine was detected at 0.3% and 0.2%, respectively. This could be expected due to the augmented molecular atoms added by curcumin and PEG in the generated Cum–PEG–BpAgNPs composite.

### 3.4 HRTEM analysis

The results of the magnified macrographs from the HRTEM instrument confirm that the particles were predominantly spherical, with a few of the particles being hexagonal, irregular, triangular, and oval (Fig. 3). The BpAgNPs image (Fig. 3A) presents polydispersed nanoparticles, with some particles overlapping each other. The PEG–BpAgNPs image (Fig. 3B) shows nanoparticles that seem suspended in a cloud with less aggregation, while the Cum–PEG–BpAgNPs image (Fig. 3C) revealed particles that are non-aggregated and uniformly arranged. This observation could signify that the core of each PEG–BpAgNP is surrounded or encapsulated by curcumin.^30^ The histogram (Fig. 3A–C) of the particle size showed that the BpAgNPs, PEG–BpAgNPs, and Cum–PEG–BpAgNPs had average sizes of 26.5, 28.4, and 27.8 nm, respectively, as computed on Image J software. In addition, the SEAD patterns of BpAgNPs, PEG–BpAgNPs, and Cum–PEG–BpAgNPs (Fig. 3D–F) showed sharp spots radiating in ring patterns indicating the analyzed samples could be crystalline.

### 3.5 XRD analysis

**Fig. 4:**
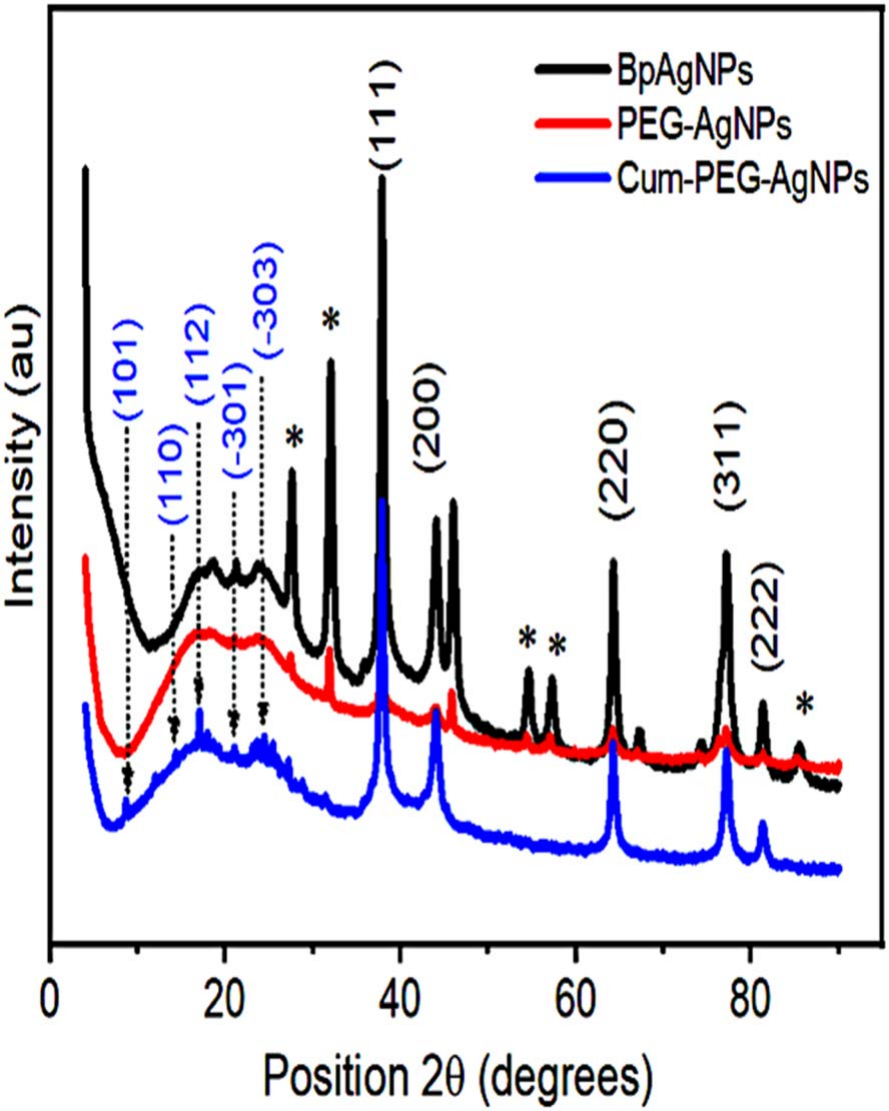
XRD peaks of BpAgNPs, PEG–BpAgNPs, and Cum–PEG–BpAgNPs.

The XRD was used to investigate the physical state of the BpAgNPs, PEG–BpAgNPs, and Cum–PEG–BpAGNPs (Fig. 4). The diffraction pattern confirmed all the formulations of BpAgNPs, PEG–BpAgNPs, and Cum–PEG–BpAgNPs were all crystalline in nature. The BpAgNPs show diffraction peaks at 38°, 44°, 64°, 77°, and 81° and correspond to the Bragg diffraction pattern for silver, with index patterns for silver at (111), (200), (220), (311), and (222). This corresponds with the ICDD library code for silver (ICDD: 04-003-7118). The diffraction peak pattern for PEG–BpAgNPs was similar to that of free silver (BpAgNPs), though the peaks were less intense. The Cum–PEG–BpAgNPs polymer composite has a stronger peak than PEG–BpAgNPs. Also, additional peaks were found on the Cum–PEG–BpAgNPs Bragg diffraction pattern and not on the BpAgNPs pattern. These additional peaks were typical of curcumin spikes at positions 8.6°, 14.4°, 17.1°, 21.1°, and 24.4°. Moreover, peaks designated with * (Fig. 4) were linked to residual organic material on the *Bidens pilosa* Ag nanoparticle material (BpAgNPs). We also note that these peaks (*) did not appear on the XRD spectra for Cum–PEG–BpAgNPs. Likewise, we observe similar peaks (*) in our previous studies,^31^ that were linked to residual active materials from the aqueous plant extract used in the biosynthesis of AgNPs from AgNO_3_.

### 3.6 *ζ*-potential analysis

**Fig. 5:**
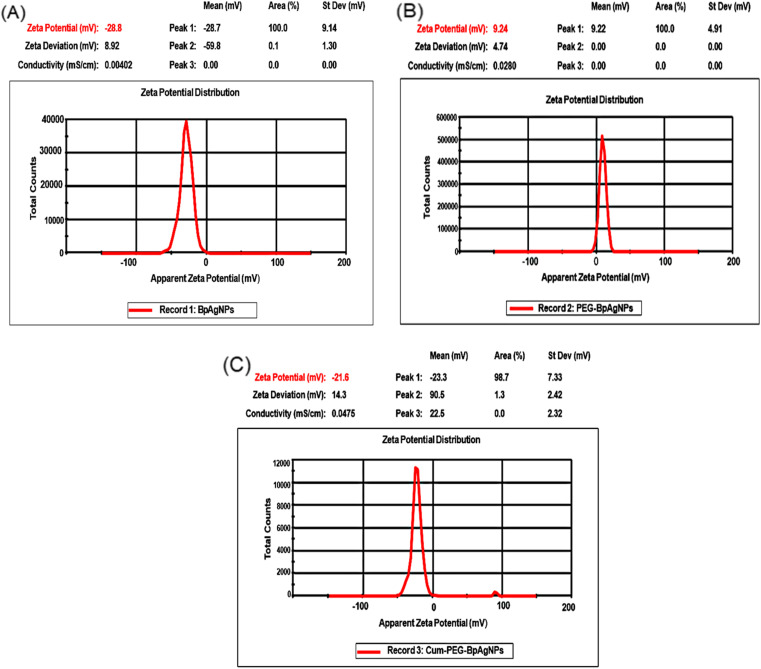
Zeta peaks of BpAgNPs, PEG–BpAgNPs, and Cum–PEG–BpAgNPs: (A) the zeta potential of BpAgNPs, (B) the zeta potential of PEG–BpAgNPs, (C) the zeta potential of Cum–PEG–BpAgNPs.

The results for the *ζ*-potential could be helpful in determining the stability of the Cum–PEG–BpAgNPs functional material. The *ζ*-potential for the bio-synthesized BpAgNPs (Fig. 5A) was −28.7 mV, and its capping with PEG led to PEG–BpAgNPs with a *ζ*-potential of 9.24 mV (Fig. 5B). Also, a *ζ*-potential of −23.3 mV was registered for the Cum–PEG–BpAgNPs (Fig. 5C).

### 3.7 Immunofluorescence of A549 SC

**Fig. 6:**
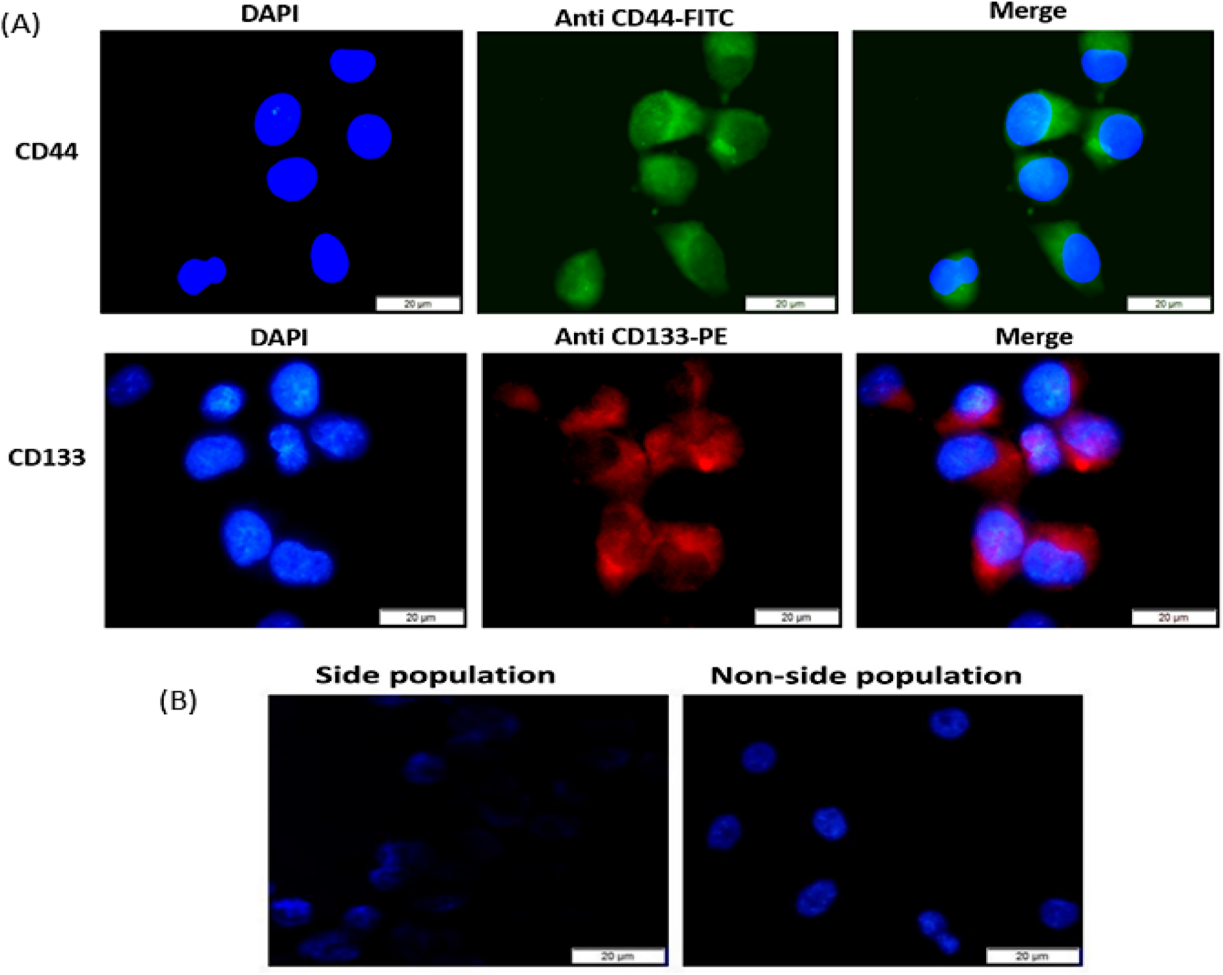
Characterization of A549 stem cells by surface makers and Hoechst 33342 dye: (A) immunofluorescence of isolated lung cancer CD44 and CD133 stem cell side population showing fluorescence of FITC stained CD44^+^ surface marker (green) and PE stained C133^+^ surface marker (red), (B) microscopic fluorescence image of A549 side population (stem cells) and A549 non-side population following Hoechst 33342 staining revealing intense fluorescence (+++) for the non-stem cell population and very low fluorescence intensity (+) by side population.

The A549 SC side population CD133^+^ and CD44^+^ isolated from the total lung cancer cells (A549) population *via* the magnetic bead separation technique were identified by immunofluorescence. The characterization *via* immunofluorescence presented positive signals for stained CD133^+^ and CD44^+^ A549 SC biomarkers. The captured image for stained anti-CD133-PE-conjugated shows CD133 positive surface markers (Fig. 6A). Also, the captured image for anti-CD44 FITC conjugate reveals the expression of CD44 antigenic surface markers. These results confirm that the pollution of cells isolated from the A549 population was actually CD133^+^ and CD44^+^ side populations (A549 SC).

### 3.8 Hoechst 33342 assay

The A549 and A549 SC were also differentiated using the Hoechst dye staining method. The intensity of Hoechst dye fluorescence taken up by the two populations was differentiated when imaging the stained slides using the DAPI filter (Fig. 6B). The intensity of fluorescence generated by the A549 side population was of low intensity (+) and that generated by the A549 non-side population presented a much brighter and more intense fluorescence (+++). This indicates the high ability of cancer stem cells to efflux the Hoechst dye.

### 3.9 Cellular uptake analysis

**Fig. 7:**
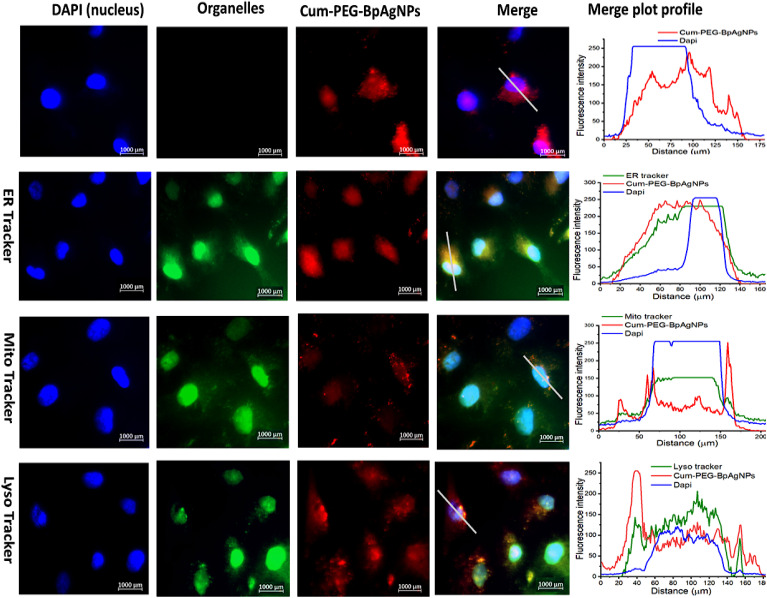
Cellular uptake fluorescence images of Cum–PEG–BpAgNPs in A549: with the DAPI stained nucleus (blue), ER, Lyso, and Mito Trackers stained the endoplasmic reticulum, lysosome, and mitochondria in green. Cum–PEG–BpAgNPs (red) localized in the endoplasmic reticulum, lysosome, mitochondria, and nucleus. The fluorescence plot profile shows co-localization of Cum–PEG–BpAgNPs, with DAPI, ER Tracker, Mito Tracker, and Lyso Tracker in A549 (nucleus and cell organelles).

**Fig. 8:**
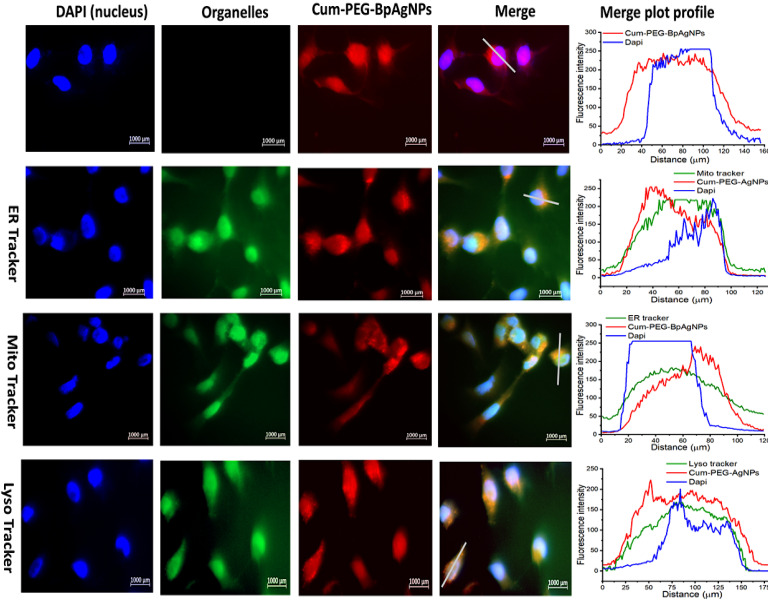
Cellular uptake fluorescence images of Cum–PEG–BpAgNPs in A549 SC: with the DAPI stained nucleus (blue), ER, Lyso, and Mito Trackers stained the endoplasmic reticulum, lysosome, and mitochondria in green. Cum–PEG–BpAgNPs (red) localized in the endoplasmic reticulum, lysosome, mitochondria, and nucleus. The fluorescence plot profile shows co-localization of Cum–PEG–BpAgNPs, with DAPI, ER Tracker, Mito Tracker, and Lyso Tracker in A549 SC (nucleus and cell organelles).

The observed fluorescence images for A549 and A549 SCs treated with Cum–PEG–BpAgNPs on the sealed are presented in Fig. 7 and 8 respectively. The merge images for A594 show the uptake of Cum–PEG–BpAgNPs in the nucleus, endoplasmic reticulum, mitochondria, and lysosome. The co-localization of Cum–PEG–BpAgNPs with the ER Tracker, Cum–PEG–BpAgNPs with the Mito Tracker, and Cum–PEG–BpAgNPs with the Lyso Tracker was observed. The merged fluorescence images for the A549 also show co-localization of Cum–PEG–BpAgNPs in the nucleus (bluish-red nucleus) as opposed to the distinctive blue nucleus when only stained with DAPI (Fig. 7). Likewise, the merged fluorescence images of Cum–PEG–BpAgNPs-treated A549 SCs confirmed the co-localization and internalization of Cum–PEG–BpAgNPs (intense fluorescence of conjugate) in the nucleus and organelles (Fig. 8). The profile plot analysis of the green, blue, and red coloration of the organelles [endoplasmic reticulum, lysosomes, and mitochondria (green)], nucleus (blue), and Cum–PEG–BpAgNPs (red) is seen in Fig. 7 and 8. The fluorescence intensity signal from the profile plots also confirms the co-localization Cum–PEG–BpAgNPs (red) with the nucleus (blue) and the organelle tracker (green) in both A549 and A549 SCs.

### 3.10 MTT assay

**Fig. 9:**
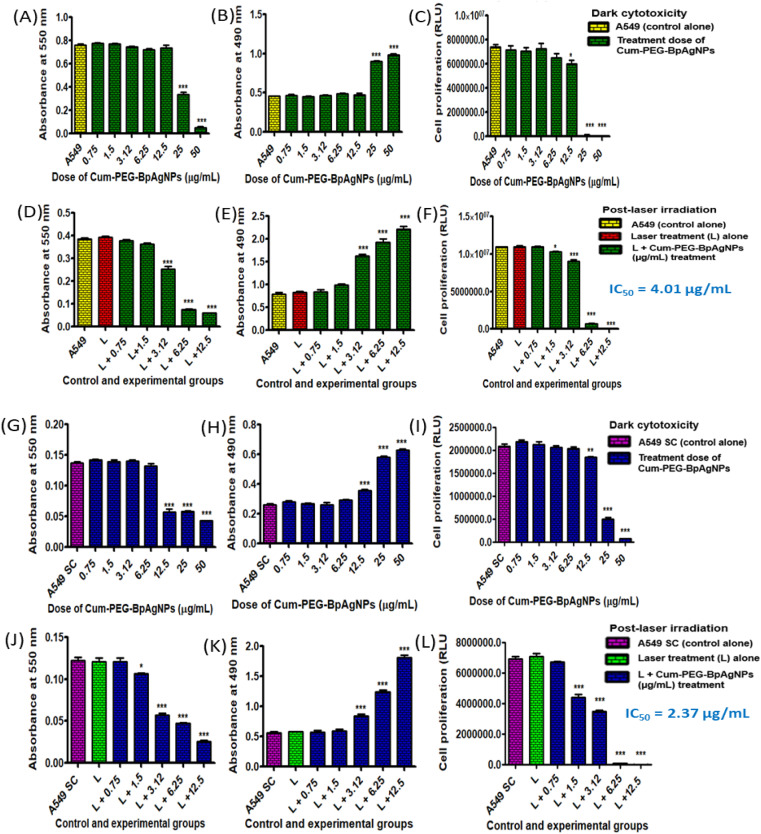
Dark cytotoxicity and post-laser irradiation dose response of Cum–PEG–BpAgNPs on A549 cells and A549 stem cells (A549 SC) using various biochemical assays with: (A) dark cytotoxicity analysis of A549 using MTT assay, (B) LDH dark cytotoxicity analysis of A549, (C) ATP dark cytotoxicity analysis of A549, (D) MTT analysis of A549 post-irradiation treatment, (E) LDH analysis of A549 post-irradiation, (F) APT analysis of A549 post-irradiation, (G) dark cytotoxicity analysis of A549 SC using MTT assay, (H) LDH dark cytotoxicity analysis of A549 SC, (I) ATP dark cytotoxicity analysis of A549 SC, (J) MTT analysis of A549 SC post-irradiation treatment, (K) LDH analysis of A549 SC post-irradiation, and (L) APT analysis of A549 SC post-irradiation. This data is analyzed for *n* = 3 per sample, using the mean ± standard error and significant changes validated at *p < 0.05, **p < 0.01, and ***p < 0.001.

The MTT assay allowed the assessment of cytotoxicity indicators linked to the viability and proliferation of A549 and A549 SC after treatment. The results reveal that Cum–PEG–BpAgNPs dark cytotoxicity concentrations from 0.75 to 12.5 μg mL^−1^ did not alter the viability of A549, and concentrations at 25 and 50 μg mL^−1^ promoted a reduction in viable cells compared to the untreated A549 (control) (Fig. 9A). The Cum–PEG–BpAgNPs-mediated laser treatment on A549 leads to an important reduction in cell viability at 3.12, 6.25, and 12.5 μg mL^−1^ (Fig. 9D). Meanwhile, the MTT dark cytotoxicity assay of Cum–PEG–BpAgNPs on the stem cells (A549 SC) showed toxicity at 12.5, 25, and 50 μg mL^−1^ and no effect from 0.75 to 6.25 μg mL^−1^ (Fig. 9G). The combined Cum–PEG–BpAgNPs and laser irradiation on A549 SC led to a reduction in cell viability at lower concentrations of the drug (1.5 to 12.5 μg mL^−1^) (Fig. 9J).

### 3.11 LDH assay

The LDH test measured the liberated amount of LDH liberated from the cytosol of A549 and A549 SC due to membrane damage following Cum–PEG–AgNPs dark cytotoxicity and the Cum–PEG–AgNPs-mediated PDT treatments. For the dark cytotoxicity studies on A549, no changes in LDH were observed for the treatment dose of Cum–PEG–AgNPs from 0.7 to 12.5 μg mL^−1^, and elevated levels of LDH were observed at 25 and 50 μg mL^−1^ (Fig. 9B). The observable dark cytotoxicity expression of LDH for the A549 SC was observed from 12.5 to 50 μg mL^−1^, and no significant changes in LDH were seen with the drug dose ranging from 0.75 to 6.25 μg mL^−1^ compared to the untreated A549 SC (Fig. 9H). On the other hand, the Cum–PEG–AgNPs mediated PDT dose induced a substantial increase in LDH at lower doses. An increase in LDH was observed with the A549 cells at 3.13, 6.25, and 12.5 μg mL^−1^, and no increase in LDH was observed for cells treated with laser only, at 0.75, and 1.5 μg mL^−1^ compared to the control (Fig. 9E). Similarly, the Cum–PEG–AgNPs-mediated PDT led to a similar result on A549 SC (Fig. 9K).

### 3.12 ATP assay

The ATP assay enabled the detection of ATP generated by cells in a milieu, and this can be correlated to proliferating cells in that milieu. The ATP dark cytotoxicity study on lung cancer cells (A549) showed great inhibition (*p* < 0.001***) when higher concentrations of Cum–PEG–BpAgNPs at 25 and 50 μg mL^−1^ were used (Fig. 9C). However, a moderate inhibition (*p < 0.05) was recorded at 12.5 μg mL^−1^, and no change in cell viability was noted when Cum–PEG–BpAgNPs at 0.75 to 6.25 μg mL^−1^ were utilized (Fig. 9C). Furthermore, Cum–PEG–BpAgNPs mediated PDT (Fig. 9F) led to a significant reduction (*p* < 0.001***) of A549 cells at 3.12, 6.25, and 12.5 μg mL^−1^. A moderate reduction (*p < 0.05) of A549 was observed post-PDT following the use of 1.5 μg mL^−1^ of the drug, and no change in viability was observed with A549 that received laser alone and Cum–PEG–BpAgNPs at 0.75 μg mL^−1^ (Fig. 9F). Similarly, the A549 SC dark cytotoxicity dose of the drug at 25 μg mL^−1^ promoted a less significant reduction (**p < 0.01) of A549 compared to the more significant reduction (***p < 0.001) of A549 at 25 and 50 μg mL^−1^ of the drug (Fig. 9I). Nonetheless, the Cum–PEG–BpAgNPs mediated PDT led to a great reduction (***p < 0.001) of A549 SC with the drug doses of 1.5, 3.12, 6.12, and 12.5 μg mL^−1^. No changes were noted for the A549 SC that received laser only and the cells that received 0.75 μg mL^−1^ of the drug plus laser treatment (Fig. 9L). In addition, the ATP data for Cum–PEG–BpAgNPs-mediated PDT data on A549 and A549 SC was utilized to determine the IC_50_ value (drug + laser IC_50_). This drug + laser IC_50_ value was validated at 4.014 μg mL^−1^ for A549 and 2.373 μg mL^−1^ for A549 SC. It should be noted that the listed concentrations (4.01 and 2.37 μg mL^−1^) had no toxicity effect on A549 and A549 SC alone. These concentrations only induced a minimum inhibition (IC_50_) when combined with laser treatment.

### 3.13 Morphology studies

**Fig. 10:**
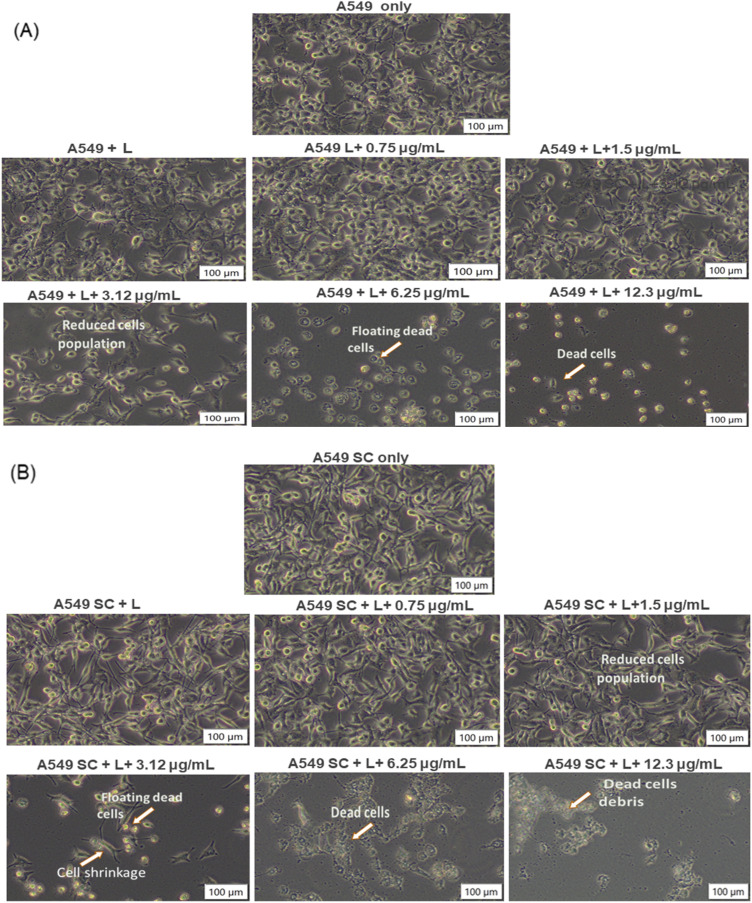
Morphology images of A549 and A459 SC post-PDT: (A) A549 cells following PDT treatment with control (A549 only), A549 treated with laser only (A549 + L), and A549 treated with laser (L) and different concentrations of the Cum–PEG–BpAgNPs (μg mL^−1^). (B) A549 SC only (lung cancer stem cells without treatment), A549 SC treated with laser only (A549 SC + L), and A549 SC treated with laser (L) and different concentrations of the Cum–PEG–BpAgNPs (μg mL^−1^). The laser light irradiation (L) treatment was performed at 5 J cm^−2^ fluency and at 470 nm.

A significant change in cell morphology was observed post-Cum–PEG–BpAgNPs-mediated PDT. Yet, no visible observed deformations or changes in the morphology of A549 were noticed at 0.75 and 1.5 μg mL^−1^ of the drug with PDT. On the other hand, the PDT treatment with drug concentrations of 3.12, 6.25, and 3.12 μg mL^−1^ led to morphological changes in cells compared to untreated A549 cells (Fig. 10A). Visible changes and deformation of A549 cells were validated by the reduction in cell number, cell shrinkage, floating cells, dead cells, and cell debris. These changes in A549 cells post-PDT (Fig. 10A) were similar to those observed with A549 SC (Fig. 10B). Nonetheless, a much lower drug concentration with PDT (1.5 μg mL^−1^ of Cum–PEG–BpAgNP plus laser) promoted a greater reduction of the A549 SC population than with the A549 cell population. Also, the morphological alterations in both A549 and A549 SC observed post-PDT were noted to be dose-dependent. More cell damage, including floating cells and dead cells, was observed when higher doses of Cum–PEG–BpAgNPs were used in combination with laser treatment.

### 3.14 ROS analysis

**Fig. 11:**
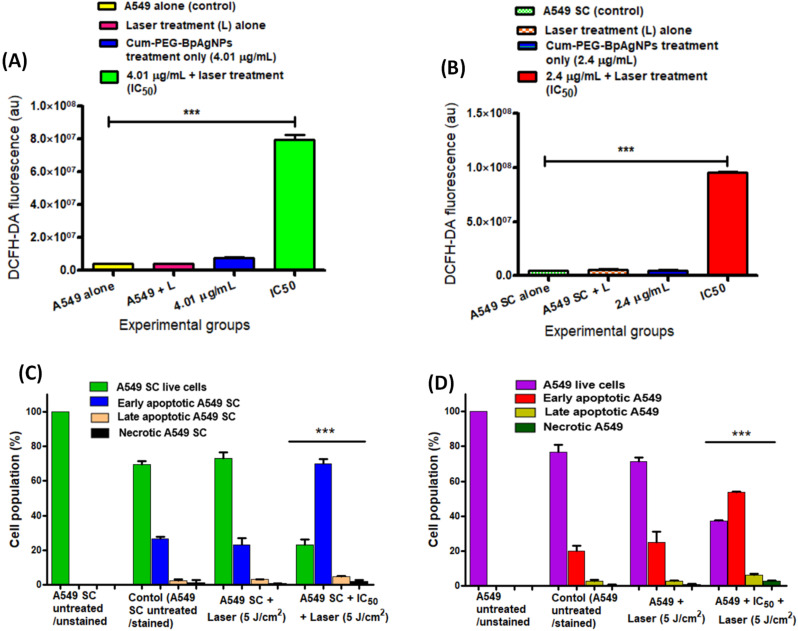
ROS and annexin V-FTIC and PI results of A549 and A549 SC post-PDT: (A) ROS produced by A549 post-PDT treatment, (B) ROS produced by A549 SC post-PDT treatment. Annexin V-FTIC and PI for Cum–PEG–BpAgNPs plus laser treatment (IC_50_) mediated against (C) A549 and (D) A549 SC. The data presented in (A)–(D) shows the mean ± standard errors for each sample at *n* = 3. The significant difference between the control and treatment groups was validated at ***p < 0.001.

We evaluated the DCFH-DA oxidation as an indicator for ROS generated by A549 and A549 SC following CUM–PEG–BpAgNPs mediated PDT. The A549 and A549 SC presented significant elevations in ROS production (***p < 0.001) following the drug and laser irradiation treatments (Fig. 11A and B respectively). The A549 and A549 SC treated with the drug (Cum–PEG–BpAgNPs) alone and laser alone did not promote any significant elevation in ROS compared to the non-treated cells. These ROS results prove the therapeutic ability of Cum–PEG–BpAgNPs as a photoactive drug.

### 3.15 Annexin and PI cell death mechanism studies

The annexin V-FITC/PI for cell death investigation *via* apoptosis and necrosis was validated. The untreated and unstained A549 and A549 SC were exploited for gating the control and experimental groups. The A549 and A549 SC treated with laser only exhibited no significant reduction or increase in the percentage of live cells compared to control groups (Fig. 11C and D). Also, more substantial apoptosis was observed than necrosis for both A549 and A549 SC following the drug and laser (IC_50_) treatment (PDT). Yet, both the A549 and A549 SC were noted to be predominantly in the early apoptotic state rather than late apoptosis. A significant reduction at ***p < 0.001 was registered when comparing the A549 control group to the PDT group. Also, a percentage reduction of A549 SC at ***p < 0.001 was recorded following PDT. The average proportion of cells at the live, early apoptosis, late apoptosis, and necrosis stages was 23.1%, 70.1%, 4.7%, and 3% for A549 SC and 37.2%, 54.7%, 6.3%, and 2.7% for A549 cells, respectively. The results indicate a percentage increase in apoptosis and a decrease in live cells in the Cum–PEG–BpAgNPs mediated PDT (IC_50_) group compared to the untreated cells and the cells that received only laser treatment.

## 4 Discussion and conclusion

Lung cancer remains a dreaded malignancy due to its associated high mortality globally. The main contributing factor to this high mortality dilemma is the inefficiency of existing therapeutic interventions such as radiation, chemotherapy, surgery, and immunotherapy.^2,3^ Therapeutic drawbacks greatly linked with this inefficiency in treatment are the involvement of LCSCs. LCSCs are sub-populations of tumor cells accountable for relapse, drug resistance, and metastasis in patients. They also show elevated resistance to lung cancer treatment interventions because of their potential to evade treatment to differentiate and self-renew.^4^ CD44^+^ and CD133^+^ LCSCs are known to be implicated in poor treatment outcomes.^4,7^ We, therefore, formulated a novel photoactive curcumin–silver nanoparticle–polymer conjugate to target cancer cells (A549) and their stem cells (A549 SC) *via* PDT. The formulated conjugate containing curcumin, BpAgNPs, and polymer (thiol polyethylene glycol amine) was confirmed *via* characterization analysis.

The UV-vis spectra confirm the formation of BpAgNPs using *Bidens pilosa* aqueous extract, which had an intense and sharp absorption at 461 nm (Fig. 1). This absorption peak is similar to that reported by Emam and Eassa, 2021 ^32^ using citrus leaves of *Citrus limon* that were stabilized using an extract of gum acacia. However, the absorption peak at 461 nm for the BpAgNP was different from that recorded for PEG–BpAgNPs (250 nm and 457 nm) and Cum–PEG–BpAgNPs (248 and 454 nm). Also, curcumin produced an absorption peak at 470 nm, which was typically very different from that generated from its conjugate (Cum–PEG–BpAgNPs), with two absorption peaks in lower wavelengths at 248 and 454 nm (Fig. 1). This could suggest an alteration in plasmonic resonance that can be linked to the strong interaction between the PEG–BpAgNPs core and outer shell of curcumin.^33^ The two peaks obtained for the Cum–PEG–BpAgNPs indicate that curcumin could have kept its diarylheptanoid chromophore functional group while bound to the PEG–BpAgNPs complex.^17^ Besides, the uncoated BpAgNPs had a border peak than that presented by PEG–BpAgNPs and Cum–PEG–BpAgNPs, which were noted to have narrow peaks. These changes in peak width are similar to the results by Kasim *et al.* 2020.^34^ These changes in peak width can be linked with the generated surface plasmon resonance which is generally influenced by the composition, morphology, shape, size, and dielectric milieu of formulated nanoparticles.^35^ The encapsulation of AgNPs by a non-functionalized PEG (polyethylene glycol) could facilitated by its hydroxyl group, which can entirely cover the surface of the AgNPs. This might be linked to the positive surface charge of AgNPs. This can equally offer stabilization to the PEG–AgNPs complex due to induced dipoles between molecules that are promoted thanks to the existence of van der Waals interactions or forces between negatively charged oxygen groups in the PEG's molecular structure and the surrounding positive charge groups on inert AgNPs surfaces.^35,36^ However, the capping of the BpAgNPs to the PEG chain in this study must have been achieved *via* thiolation and not *via* the hydroxyl group, considering that the type of PEG we utilized was a thiol polyethylene glycol amine (linear heterobifunctional PEG).^37^

The FTIR spectroscopy analysis was done to identify the active molecules involved in the synthesis of BpAgNPs, PEG–BpAgNPs, and Cum–PEG–BpAgNPs. The absorption spectra (Fig. 2A) for Bp aqueous extract have a peak at 3414 cm^−1^ which could be due to O–H vibration, which may originate from the plant's secondary metabolites like amino acids and polyphenols. These metabolites may be responsible for the bio-reduction, chelation, and even capping of nanoparticles. The peak at 2827 cm^−1^ indicates vibration from the C–H bond. The peak at 1597 cm^−1^ can originate from a C=O group conjugated with a double bond or from an amide that is responsible for the bio-reduction of AgNO_3_ to the AgNPs. It can also be assigned to C=C vibration stretching originating from unsaturated metabolites from the plant extract, like terpenoids. Likewise, the peak at 1387 cm^−1^ corresponds with the C–H bend.^38^ The peak at 1070 is attributed to the C–OH stretch. Minor peaks around 770 cm^−1^ are linked with vibration stretching from C=C bending.^38^ Peaks at 614 and 534 cm^−1^ indicate C–Cl vibration from alkyl halides or phenyl groups.^31^

The PEG (HS-PEG2K-NH_2_) intense peaks (Fig. 2B) noted to be involved in coating the free BpAgNPs appeared at 3436 cm^−1^. This indicates NH vibrations from an amine or from the hydroxyl group. A peak was noted at 2888 cm^−1^ revealing a C–H vibration stretch. The C–H aliphatic stretch was revealed due to peaks at 1348 cm^−1^ indicating vibrations originating from the C–H bend. The peak at 1282 cm^−1^ was for C–O vibrations, and at 1107 cm^−1^ for a C–OH stretching or from a C–O–C from an ether linkage.^35,36,39^ The band for PEG at 842 and 526 cm^−1^ could be radiating from the C–S and H–S associated with thiol at the end of the PEG chain^40,41^ and can be responsible for affinity binding with free BPAgNPs. Comparing the free BpAgNPs and PEG–BpAgNPs (Fig. 2B). We noticed some shift in peaks to lower wavelengths as follows; 3434 and 3426 cm^−1^, 2924 to 2921 cm^−1^, 2076 to 2066 cm^−1^, 1091 to 1083 cm^−1^, and 602 to 581 cm^−1^. In addition, a more intense peak at 2850 cm^−1^ was perceived for PEG–BpAgNPs and not for the free BpAgNPs. These results confirm the molecular re-arrangement of the free BpAgNPs atoms in the PEG–BpAgNPs nanopolymer. Also, the shift in peak from 602 (in free BpAgNPs) to 581 cm^−1^ (in PEG–BpAgNPs) could be due to the involvement of the S–H group noted at 526 cm^−1^ on the PEG spectra. This indicates PEGylation of BpAgNPs which must be achieved *via* thiolation interactions where the thiol (H–S) on the PEG polymer (a linear heterobifunctional HS-PEG2K-NH_2_ polymer) binds to the free BpAgNPs and the amine (NH_2_) side of the polymer remains exposed to the surface.^42^ The thiolation of free AgNPs can be possible *via* chemisorption interactions, where the surface binding of the ligand with nanoparticles leads to electron density donation from the ligand (functional group) to metal nanoscale material. Still, reactions, including redox and electrochemical reactions, may promote the interaction of thiol with AgNPs. Besides, the effect of a chemical group substituent or replacement is also noted regarding the formation of thiolates of polymeric silver.^43,44^ The thiolation of AgNPs can lead to the generation of different compounds depending on the type of thiolate used.^43^ Ag_2_S and AgS-R forms of complexes were confirmed following the addition of organothiols during the synthesis of AgNPs.^45^ Thiol shows a strong affinity for metal, especially silver, and has been exploited in various analytical sensors. Also, thiols like cysteine, mercaptohexanol, glutathione, cysteine, and cysteamine thioglycolic acid can be utilized in the capping of AgNPs to stabilize and stop them from aggregating.^43,46^ Moreover, functional groups like thiol and amine can enable the surface functionalization of metallic nanoparticles like silver into more solid support, forming conjugated biomolecules and hybrid materials.^42^

**Fig. 12:**
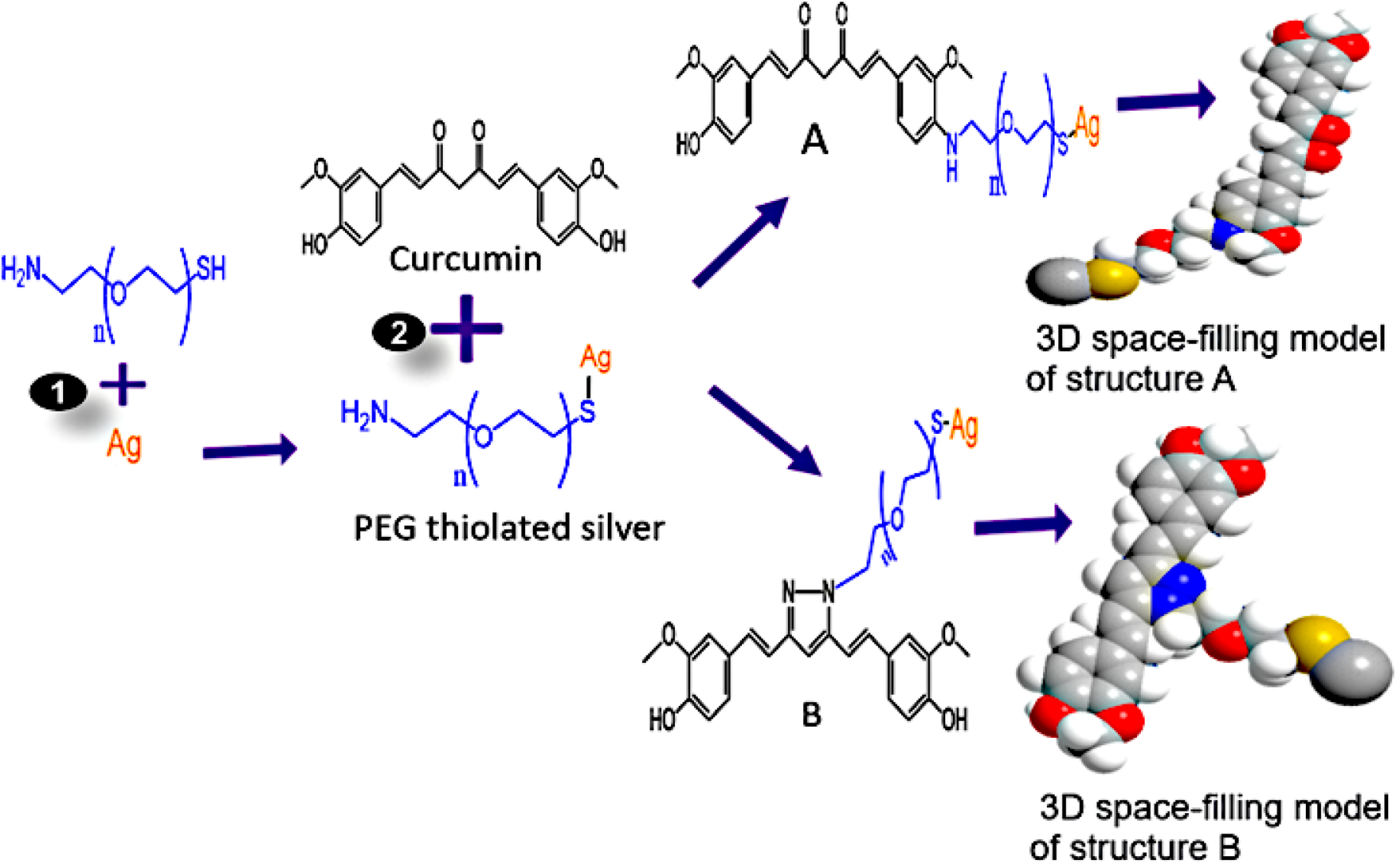
Proposed structures of Cum–PEG–BpAgNPs: reaction (1) involves the sonication of Ag (BpAgNPs) in PEG, followed by 24 h incubation at room temperature in the dark; in reaction (2), curcumin was sonicated in PEG thiolated silver (PEG–BpAgNPs), followed by incubation in the dark at 4 °C for 24 h. A and B represent the possible structures of curcumin–silver nanoparticle–polymer conjugate (Cum–PEG–BpAgNPs) formed.

Curcumin (Fig. 2C) had an intense peak at 3435 cm^−1^ that was linked with vibration from a hydroxyl group, possibly from phenolic vibration stretching. Peaks at 2974 and 2853 cm^−1^ are from an asymmetric vibration from the –CH_3_ and –CH_2_ groups, respectively. The peaks at 1628 cm^−1^ are linked to C=O vibration. While peaks at 1509 and 1429 cm^−1^ are attributed to stretching of vibration from C=C in the curcumin benzene ring and C–H radiating from an olefinic bend bounded by curcumin's benzene ring, respectively.^37^ Absorption at 1384 cm^−1^ is from CH_3_ bending. In addition, peaks at 1275 and 1150 cm^−1^ indicate vibration stretching from a C–O. Peaks at 1032 and 964 cm^−1^ are matched with vibration from a C–OH group.^47,48^ Peaks at 887 and 808 cm^−1^ are assigned with C–O and C–H vibrations that stretch out of the aromatic ring plane of curcumin.^49^ However, curcumin's conjugation with PEG–BpAgNPs to Cum–PEG–BpAgNPs (Fig. 2C) led to shifts in absorption peaks when comparing the PEGylated silver nanoparticles (PEG–BpAgNPs) and its curcumin conjugate (Cum–PEG–BpAgNPs) (shift in peaks from 3425 to 3423 cm^−1^, 2921 to 2929 cm^−1^, 1631 to 1612 cm^−1^, 1083 to 1021 cm^−1^, and 581 to 554 cm^−1^). Similarly, new intense peaks were noted on the Cum–PEG–BpAgNPs absorption spectra at 1499, 1263, and 819 cm^−1^ which were not non-prominent on the PEG–BpAgNPs spectra. This re-arrangement or shift in peaks in this scenario indicates the conjugation of curcumin with PEG–BpAgNPs. Curcumin can take part in various chemical reactions such as hydrogen donation, nucleophilic reactions, Michael reactions, enzymatic reactions, degradation, and hydrolysis reactions.^50^ Thus, we proposed the possible structures for the curcumin conjugate (Fig. 12). Hamed *et al.* (2013) report the formation of an amine-modified magnetite nanocomposite that is efficiently conjugated with curcumin *via* its amine-active side.^51^ Also, the formation of curcumin-based diazepine, amine, and isoxazoles that were produced *via* curcumin's reaction with different forms of amines was documented.^52^

HRTEM analysis was done to determine the size and morphology of BpAgNPs, PEG–BpAgNPs, and Cum–PEG–BpAgNPs. Similar morphological presentations, including spherical (predominant shape), hexagonal, irregular, triangular, and oval shapes, were obtained for BpAgNPs, PEG–BpAgNPs, and Cum–PEG–BpAgNPs. The average particle size for PEG–BpAgNPs and Cum–PEG–BpAgNPs was slightly increased compared to the free BpAgNPs (Fig. 3A–C). Ibraheem *et al.* (2012) reported similar results.^53^ Moreover, the conjugate Cum–PEG–BpAgNPs show no aggregation compared to the BpAgNPs and PEG–AgNPs, while the PEG–BpAgNPs were less aggregated than the BpAgNPs. This shows that PEG could prevent nanoparticles from aggregating. This can be linked to the steric effects and space or distance between nanoparticles.^53,54^

The XRD confirmed the production of a crystalline Cum–PEG–BpAgNPs, which is observed to have stronger diffraction peaks than PEG–BpAgNPs (Fig. 4). This suggests that the molecular arrangement of atoms in the Cum–PEG–BpAgNPs complex was more favored than in PEG–BPAgNPs.^55^ Moreover, similar XRD peak positions are presented by BpAgNPs and PEG–BpAgNPs. This suggests the non-modification of the BpAgNPs components after PEGylation.^56^ Yet, weaker peaks were visible in PEG–BpAgNPs XRD spectra than with BpAgNPs spectra. This confirms a strong thiolation interaction of the polymer (HS-PEG2K-NH_2_) with BpAgNPs.

The *ζ*-potential descriptor validates the electrostatic potential in the shear plane exiting between nanoparticles and the solvent suspension for those nanoparticles and thus provides knowledge on the particle's stability and surface coating.^54^ BpAgNPs, PEG–BpAgNPs, and Cum–PEG–BpAgNPs were tested for their surface charge and stability by *ζ*-potential analysis. Values of *ζ*-potential in the range ±0–10, ±10–20, ±20–30, and >±30 mV are considered unstable, relatively unstable, fairly stable, and highly stable, respectively.^34^ Similarly, charges higher than >±10 mV are connoted to exhibit stronger inter-particle repulsion.^54^ The −28.7 and −23.3 mV *ζ*-potential recorded for BpAgNPs and Cum–PEG–BpAgNPs in this study suggest they were fairly stable. Also, the PEG–BpAgNPs had a positive charge of +9.24 mV. This could be linked with the exposed surface net charge of the amine groups on PEG–BpAgNPs associated with the PEG [thiol polyethylene glycol amine (HS-PEG2K-NH_2_)] that was utilized in BpAgNPs PEGylation. Polyethylene glycol polymers often work as stabilizers, thus restricting Ag ion mobility and ensuring the non-agglomeration of prepared nanoparticles.^53^ However, the HS-PEG2K-NH_2_ interaction with free BpAgNP may have been favored *via* the thiol (HS) group while allowing the NH_2_ group at the free end of the PEG–BpAgNPs polymer. This is linked to the high-affinity binding of thiol with metals.^46^ Besides, the amine-functional group at the outer surface of the formulated PEG–BpAgNPs polymer could be responsible for the positive *ζ*-potential (+9.24 mV) of the polymer (Fig. 5B). These results are similar to those reported by Lee and Jun (2019) where the interaction of AgNPs with polyethyleneimine resulted in the formation of a polymer conjugate with a positive charge.^57^

Furthermore, we isolated and characterized the CD133^+^ and CD44^+^ A549 SC for *in vitro* PDT. The CD133 and CD44 CS (cancer stem cells) markers are the most identified biomarkers in lung cancer.^58^ The CD44 markers are transmembrane glycoproteins that can bind with a predominant stem cell polysaccharide hyaluronic acid, to facilitate differentiation, migration, homing, and adhesion within CS. CD44 markers are key players in CSC identification since they control the processes of differentiation, adhesion, migration, and homing.^58^ In lung cancer prototypes, increased expression of CD44 was first reported in squamous metaplasia, activated type II pneumocytes, and NSCLC indicating that it might be involved in the progression of the illness.^59^ The CD44^+^ subpopulation of NSCLC is capable of forming spheroid bodies and initiating the formation of tumors *in vivo*. Likewise, CD44^+^ cells are also able to express pluripotency genes such as NANOG, OCT4, and SOX2, which are not expressed in CD44-cells. Moreover, CD44^+^ cells are noted to be resistant to anti-cancer drugs like cisplatin.^60^ The CD133 (Prominin-1) markers are defined as the five-transmembrane glycoproteins on the cell surface.^61^ CD133^+^ cells show resistance to cancer chemotherapy and exhibit large amounts of ATP-binding cassette G2, which indicates their SP phenotype. CD133^+^ cells have also been linked with the short survival of patients with NSCLC who took platinum-based treatment. This highlights the correlation between the CD133^+^ cells' involvement in the poor efficiency of lung cancer chemotherapy.^58^ In addition, CD133^+^ cells are noted to produce genes that take part in stemness, motility, drug efflux, and adhesion.^61^ We, therefore, isolated CD44 and CD133 A549 SC in this study. The immunofluorescence results (Fig. 6A) confirmed CD133^+^ surface markers that are stained with the anti-CD133 PE-conjugated (red). The CD44^+^ surface marker was revealed thanks to the surface binding of anti-CD44 FITC conjugate with the CD44^+^ marker, which was visualized as green fluorescence (Fig. 6A). The results were supported by the Hoechst 33342 analysis (Fig. 6B). The Hoechst 33342 dye for cancer stem cell identification helps to define this type of cell population by their capacity to actively pump out the Hoechst dye from the cell nucleus (Chizenga *et al.*, 2019; Mkhobongo *et al.*, 2022).^62,63^ The dye binds actively to the DNA minor groove at the AT-rich areas, and its fluorescence intensity will vary depending on the chromatin structure, the stage at which the cell is in the cell cycle, and the DNA content. The cellular uptake of lipophilic dyes, including Hoechst 33342, is universal.^64,65^ However, the potential to efflux or pump out the dye is more restricted to the stem cell subpopulation nucleus.^62,63^ Though the molecular principal for this assay is still uncertain, the efflux mechanism is attributed to the uptake of Hoechst 33342 by ATP-binding cassette transporter proteins [ABCG1a/b (Mdr1a/b) and ABCG2 (Bcrp1)].^66^ The Hoechst 33342 analysis in this work shows low fluorescence intensity for the side population (A549 SC) as opposed to the intense fluorescence intensity of the non-side population (Fig. 6B). The reason for the low Hoechst fluorescence in the side population is that ATP-binding cassette transporter proteins pump out the Hoechst dye from the cell nucleus. The ABC transmembrane protein transporters can attach to ATP, causing its hydrolysis. Likewise, these proteins can function as channels, receptors, and multidrug transporters, thus enabling cells to eliminate different endogenous compounds and cytotoxic harmful agents using ATP. These cytotoxic agents include chemotherapeutic drugs, which imply the involvement of cancer stem cell (CSC) transporter ABC proteins in drug resistance.^62,66^

The biochemical assays, including MTT, LDH, and ATP assays, confirmed the toxicity of the Cum–PEG–BpAgNPs on A549 and A549 SC. These assays showed elevated toxicity of the Cum–PEG–BpAgNPs on treated cells when compared to the cells that did not receive treatment. The toxicity of Cum–PEG–BpAgNPs was noticed to increase when higher concentrations were used for cell treatment (Fig. 9). Also, the data from the dark cytotoxicity study using the biochemical assays led us to determine the Cum–PEG–BpAgNPs dose with minimal toxicity that was exploited in the PDT study. The dark cytotoxicity study using MTT and LDH assays showed no toxicity on A549 at Cum–PEG–BpAgNPs doses ranging from 0.75 to 12.5 μg mL^−1^ (Fig. 9A and B). In contrast, the ATP assay indicated that Cum–PEG–BpAgNPs induce no toxicity on A549 from 0.75 to 6.25 μg mL^−1^ (Fig. 9C). Also, the drug dark cytotoxicity study on A549 SC showed no toxicity from 0.75 to 6.25 μg mL^−1^ when the MTT, LDH, and ATP assays were performed (Fig. 9G–I). However, significant dark toxicity (***p < 0.001) was recorded at 12.5 to 50 μg mL^−1^ (Fig. 9G–I) after the A549 SC treatment. These results show that the drug at lower doses (from 0.75 to 6.25 μg mL^−1^) was non-toxic to the A549 SC. In contrast, the drug was non-toxic to A549 from 0.57 to 12.5 μg mL^−1^. Although the effect of Cum–PEG–BpAgNPs was not investigated on normal cells in this study, reports indicated that curcumin nanoconjugates have demonstrated encouraging outcomes in lowering toxicity to normal cells and specifically targeting cancer cells.^67^ Similarly, a nanoconjugate of curcumin with serum albumin of human origin (HAS–curcumin nanoparticles) exhibited minimal toxicity in normal cells while showing improved and selective toxicity in cancer cells.^68^ This selectivity in toxicity is further supported by a study that indicated that PLA-HA/Fe_3_O_4_–curcumin nanoparticles exhibited a significant cytotoxic impact on colorectal cancer but with negligible toxicity on non-cancerous cells.^69^ In the PDT study, Cum–PEG–BpAgNPs at 0.57 to 12.5 μg mL^−1^ were harmonized for A549 and A549 SC PDT treatment, even though the drug at 12.5 μg mL^−1^ induced minimal toxicity (*p < 0.05) in A549 SC. The Cum–PEG–BpAgNPs-mediated PDT led to cytotoxicity in A549 (Fig. 9D–F) and A549 SC (Fig. 9J–L). The Cum–PEG–BpAgNPs in the mediation of PDT thus demonstrated to be very photoactive due to its induced cytotoxicity on both A549 and A549 SC. The IC_50_ recorded was 4.014 and 2.372 μg mL^−1^ for A549 and A549 SC, respectively.

In addition, the results obtained using the biochemical assays correlated with the morphological alteration observed in the treated cells treated post-PDT (Fig. 10A and B). Alterations, including shrinkage, floating, and dead cells, were observed post-PDT. The alteration in cells was more dose-dependent, as an increase in drug concentration with PDT led to more cell damage and even cell death. These morphological alterations are similar to those presented by cancer cells that go through oxidative stress and, hence, apoptosis.^70^ These prove that the formulated Cum–PEG–BpAgNPs is an effective drug and can also be exploited in lung cancer PDT treatment since it can target both the lung cancer cell (A549) and their stem cells (A549 SC). However, research on conjugating naturally PS such as curcumin with green AgNPs for the photoactive targeting of both cancer cells and their stem cells *via* PDT seems lacking. Generally, nanoparticles that are designed as carriers for drugs like PSs can enhance the PDT effect through active and passive targeting strategies. The nanoparticle carriers that are intended for cancer active targeting therapy are also modified by attaching to them active moieties such as aptamers, peptides, and antibodies to enhance the specificity of cell targeting by the PS molecule.^71^ Nonetheless, this was not the case in this study, since the surface modification of nanocarrier (BpAgNPs) was achieved *via* PEGylation for passive targeting. In passive targeting, the nanocarrier containing the drug can easily penetrate to accumulate in the tumor tissue thanks to poor lymphatic outflow and leaky vasculature. This is enhanced permeability and retention (EPR) and is greatly linked with passive targeting treatment in cancer.^71,72^ The unique nature of the tumor microenvironment which is atypical to normal healthy cells also facilitates the easy deposition of PSs when these PSs are designed as nanoparticles. Besides, the microvasculature of the tumor and the inherent factors of nanoparticles such as shape, size, and *ζ*-potential also contribute to facilitating the delivery of nanoparticles in passive targeting.^71–73^ This passive targeting of the tumor cell with nano-formulated drugs like PSs is vital for PDT. Mohammadi *et al.* (2020) confirmed the formulation of a curcumin nano-polymer drug (selenium–polyethylene glycol–curcumin) that was activated at 100 μg mL^−1^ with laser irradiation to induce toxicity in melanoma cancer Mohammadi.^74^ Fadeel *et al.* (2020) produced a PEGylated nanocarrier filled with curcumin that showed improved cytotoxicity on the human skin cancer cell line (A431) at post-PDT.^75^ Reasons for the improved cytotoxicity were linked to the enhanced solubility of curcumin by the nanocarriers, better permeability, improved cellular uptake, and elevated surface area.^75^ This is also supported by the results of this study which validate an improved cellular uptake of Cum–PEG–BpAgNPs in A549 and A549 SC (Fig. 7 and 8). The laser light treatment can also markedly boost cytotoxicity *via* ROS production if an effective cellular uptake of the PS (drug) takes place. The cytotoxic intracellular ROS then incites cellular organelle damage, compromising mitochondrial membrane function and resulting in apoptosis.^75^

Generally, PDT events are coupled with ROS generation, which greatly influences the therapeutic efficacy.^76,77^ The activation of ROS following PDT treatment can cause the oxidation of deacetylated DCFDA producing fluorescent DFC which correlates with the amount of cellular ROS.^76,78^ Another study found that curcumin-loaded nanoPLGA polymers led to the greatest production of ROS compared to the isomolar form of curcumin, while no ROS was generated by the untreated cells. They associated this improved PDT ROS production with increased facilitated endocytic internalization of the curcumin nanodrug polymer.^79^ Also, studies on curcumin-encapsulated chitosan/tripolyphosphate nanodrug indicated that the drug was able to significantly activate ROS production in epidermal growth factor overexpressed cancer cells (MKN45).^80^ These results are similar to those obtained in this study, which showed a significant (***p < 0.001) elevation in ROS in both A549 and A549 SC after the Cum–PEG–BpAgNPs and laser light treatment. The elevated cytotoxic intracellular ROS can incite cellular organelle damage, compromising the mitochondrial membrane function.^75^ As a result, the mitochondrial matrix swells osmotically and the transmembrane potential of the mitochondria is immediately lost. A cessation of mitochondrial energy production will occur, due to the non-exchange of essential molecules between the mitochondria matrix and the cytoplasm and finally cell apoptosis.^76^ The apoptosis process is vital in cell homeostasis and different biochemical and morphological alterations are linked to this process. The apoptotic mechanism was investigated using the annexin V-FITC/PI assay. The assay enables the determination of non-apoptotic and apoptotic cell populations at a particular state. The Cum–PEG–BpAgNPs mediated PDT induced significant (***p < 0.001) apoptosis. A high percentage of the A549 and A549 SC were detected at the apoptosis state (Fig. 11C and D). This shows that the cell death pathway should have been *via* apoptosis. This result also supports the ability of Cum–PEG–BpAgNPs as an efficient PS for ROS generation in PDT, since the efficacy of PDT in different model systems is founded on ROS formation, which acts as a key regulator for active cell death.^81^ Nonetheless, PDT can stimulate both apoptosis and necrosis in treated cells. Apoptosis is regarded as an active, regulated, and energy-needing event, while necrosis is an entropic process that results as a consequence of membrane integrity damage and impaired metabolic homeostasis.^82^

This study therefore produced a novel conjugate (Cum–PEG–BpAgNPs) using green synthesized BpAgNPs and curcumin. The combination of this molecule with PDT showed great cytotoxicity efficacy by eliminating lung cancer cells and their stem cells. The Cum–PEG–BpAgNPs molecule could be exploited as an alternative PS in PDT to initiate ROS and hence apoptosis in most lung cancer cells that show resistance to conventional treatment.

## Data availability

The authors confirm that the data supporting the results are available in this article. The datasets obtained and analyzed during the current study can be available from the corresponding author at reasonable request.

## Author contributions

G. K.: conception and design of the study, laboratory work, acquisition of data, analysis and interpretation of data and drafting of the article. R. C. and H. A.: supervision of study, writing, revision of content, editing, final approval of the version to be published and corresponding author.

## Conflicts of interest

The authors declare that they have no conflicts of interest to report regarding the present study.

## Supplementary Material

RA-015-D4RA06035K-s001
