# Supplementary material for: Green silver nanoparticles curcumin conjugate induced photodynamic therapy of lung cancer and lung cancer stem cells
Source: RSC Adv. 2025 Feb 14;15(7):5020–41. doi: 10.1039/d4ra06035k (PMC11827557; doi:10.1039/d4ra06035k)
Supplement: RA-015-D4RA06035K-s001 [file RA-015-D4RA06035K-s001.pdf]

Supplementary Information (SI) for RSC Advances.
This journal is © The Royal Society of Chemistry 2025

**1. Biochemical assay (ATP, MTT, and LDH) raw data following dark cytotoxicity and PDT treatments on A549 and A549 SC using Cum-PEG-BpAgNPs (Fig. 9)**

| | | | | | | | | |
|---|---|---|---|---|---|---|---|---|
| **Cum-PEG-BpAgNPs A549 dark cytotoxicity data for ATP, MTT, and LDH** | | | | | | | | |
| **ATP** | **A549 (contro)** | **0,75 µg/mL** | **1,5 µg/mL** | **3,12 µg/mL** | **6,25 µg/mL** | **12,5 µg/mL** | **25 µg/mL** | **50 µg/mL** |
| | 7759885 | 6604042 | 6630694 | 8094162 | 5916187 | 5754382 | 198033 | 14044 |
| | 7437416 | 7013580 | 6829483 | 7057626 | 6780575 | 6632148 | 18232 | 27354 |
| | 7080829 | 7859016 | 7700524 | 6612681 | 6888897 | 5621851 | 17493 | 13672 |
| | | | | | | | | |
| **MTT** | **A549 (control)** | **0,75 µg/mL** | **1,5 µg/mL** | **3,12 µg/mL** | **6,25 µg/mL** | **12,5 µg/mL** | **25 µg/mL** | **50 µg/mL** |
| | 0,753 | 0,77 | 0,778 | 0,753 | 0,712 | 0,68 | 0,352 | 0,04 |
| | 0,742 | 0,784 | 0,772 | 0,742 | 0,745 | 0,762 | 0,3 | 0,058 |
| | 0,783 | 0,767 | 0,767 | 0,749 | 0,698 | 0,764 | 0,362 | 0,059 |
| | | | | | | | | |
| **LDH** | **A549 (control)** | **0,75 µg/mL** | **1,5 µg/mL** | **3,12 µg/mL** | **6,25 µg/mL** | **12,5 µg/mL** | **25 µg/mL** | **50 µg/mL** |
| | 0,466 | 0,461 | 0,463 | 0,477 | 0,509 | 0,503 | 0,9 | 0,993 |
| | 0,444 | 0,497 | 0,436 | 0,448 | 0,479 | 0,436 | 0,92 | 0,952 |
| | 0,455 | 0,452 | 0,456 | 0,473 | 0,482 | 0,491 | 0,891 | 1,001 |
| | | | | | | | | |
| | | | | | | | | |
| **Cum-PEG-BpAgNPs plus laser light A549 (A549 PDT) data for ATP, MTT, and LDH** | | | | | | | | |
| **ATP** | **A549 (control)** | **A549 + L (Light)** | **L + 0,75 µg/mL** | **L + 1,5 µg/mL** | **L + 3,12 µg/mL** | **L + 6,25 µg/mL** | **L + 12,5 µg/mL** | |
| | 10829880 | 10732600 | 11023020 | 10478420 | 9002954 | 699146 | 36777 | |
| | 11010190 | 11229610 | 10997290 | 10147160 | 9399619 | 743577 | 40206 | |

| | | | | | | | | |
|---|---|---|---|---|---|---|---|---|
| | 10946280 | 10938200 | 10830700 | 10130780 | 8721033 | 681136 | 38510 | |
| | | | | | | | | |
| **MTT** | **A549 (control)** | **A549 + L (Light)** | **L + 0,75 µg/mL** | **L + 1,5 µg/mL** | **L + 3,12 µg/mL** | **L + 6,25 µg/mL** | **L + 12,5 µg/mL** | |
| | 0,392 | 0,386 | 0,378 | 0,357 | 0,243 | 0,075 | 0,062 | |
| | 0,388 | 0,388 | 0,388 | 0,373 | 0,241 | 0,069 | 0,061 | |
| | 0,377 | 0,403 | 0,365 | 0,362 | 0,276 | 0,081 | 0,06 | |
| | | | | | | | | |
| **LDH** | **A549 (control)** | **A549 + L (Light)** | **L + 0,75 µg/mL** | **L + 1,5 µg/mL** | **L + 3,12 µg/mL** | **L + 6,25 µg/mL** | **L + 12,5 µg/mL** | |
| | 0,782 | 0,852 | 0,814 | 0,962 | 1,615 | 2,068 | 2,127 | |
| | 0,75 | 0,774 | 0,935 | 0,935 | 1,558 | 1,917 | 2,143 | |
| | 0,848 | 0,848 | 0,762 | 1,055 | 1,691 | 1,804 | 2,356 | |
| | | | | | | | | |
| | | | | | | | | |
| **Cum-PEG-BpAgNPs A549 stem cell (A549 SC) dark cytotoxicity data for ATP, MTT, and LDH** | | | | | | | | |
| **ATP** | **A549 SC (control)** | **0,75 µg/mL** | **1,5 µg/mL** | **3,12 µg/mL** | **6,25 µg/mL** | **12,5 µg/mL** | **25 µg/mL** | **50 µg/mL** |
| | 2126561 | 2227508 | 2019991 | 2115021 | 1994532 | 1846036 | 583657 | 70567 |
| | 2011094 | 2213990 | 2213980 | 2096432 | 1994532 | 1883286 | 464951 | 83030 |
| | 2139966 | 2142887 | 2147288 | 1996329 | 2111621 | 1829263 | 442321 | 77151 |
| | | | | | | | | |
| **MTT** | **A549 SC (control)** | **0,75 µg/mL** | **1,5 µg/mL** | **3,12 µg/mL** | **6,25 µg/mL** | **12,5 µg/mL** | **25 µg/mL** | **50 µg/mL** |
| | 0,133 | 0,144 | 0,136 | 0,14 | 0,136 | 0,053 | 0,061 | 0,043 |
| | 0,139 | 0,141 | 0,1382 | 0,136 | 0,124 | 0,053 | 0,056 | 0,043 |
| | 0,14 | 0,141 | 0,144 | 0,143 | 0,137 | 0,066 | 0,057 | 0,042 |
| | | | | | | | | |
| **LDH** | **A549 SC (control)** | **0,75 µg/mL** | **1,5 µg/mL** | **3,12 µg/mL** | **6,25 µg/mL** | **12,5 µg/mL** | **25 µg/mL** | **50 µg/mL** |
| | 0,266 | 0,296 | 0,259 | 0,241 | 0,289 | 0,359 | 0,598 | 0,63 |

| | | | | | | | | |
|---|---|---|---|---|---|---|---|---|
| | 0,241 | 0,264 | 0,265 | 0,249 | 0,294 | 0,344 | 0,58 | 0,61 |
| | 0,272 | 0,28 | 0,277 | 0,293 | 0,298 | 0,368 | 0,563 | 0,645 |
| | | | | | | | | |
| | | | | | | | | |
| **Cum-PEG-BpAgNPs plus laser light A549 SC (A549 SC PDT) data for ATP, MTT, and LDH** | | | | | | | | |
| **ATP** | **A549 SC (control)** | **A549 SC + light (L)** | **L + 0,75 µg/mL** | **L + 1,5 µg/mL** | **L + 3,12 µg/mL** | **L + 6,25 µg/mL** | **L + 12,5 µg/mL** | |
| | 7056351 | 7502543 | 6747626 | 4761302 | 3373367 | 84560 | 16613 | |
| | 6605690 | 6790123 | 6680915 | 4299484 | 3407340 | 77420 | 15989 | |
| | 7104693 | 6945013 | 6763211 | 4194422 | 3656078 | 79805 | 14669 | |
| | | | | | | | | |
| **MTT** | **A549 SC (control)** | **A549 SC + light (L)** | **L + 0,75 µg/mL** | **L + 1,5 µg/mL** | **L + 3,12 µg/mL** | **L + 6,25 µg/mL** | **L + 12,5 µg/mL** | |
| | 0,119 | 0,121 | 0,129 | 0,107 | 0,058 | 0,047 | 0,023 | |
| | 0,13 | 0,112 | 0,12 | 0,105 | 0,06 | 0,049 | 0,026 | |
| | 0,118 | 0,129 | 0,114 | 0,108 | 0,053 | 0,046 | 0,028 | |
| | | | | | | | | |
| **LDH** | **A549 SC (control)** | **A549 SC + light (L)** | **L + 0,75 µg/mL** | **L + 1,5 µg/mL** | **L + 3,12 µg/mL** | **L + 6,25 µg/mL** | **L + 12,5 µg/mL** | |
| | 0,593 | 0,583 | 0,518 | 0,585 | 0,91 | 1,267 | 1,89 | |
| | 0,541 | 0,589 | 0,582 | 0,643 | 0,82 | 1,184 | 1,749 | |
| | 0,561 | 0,57 | 0,62 | 0,537 | 0,794 | 1,28 | 1,786 | |

## 2. ROS data following treatment of A549 and A549 SC $IC_{50}$ doses (Fig.11 A and B)

| **ROS data following Cum-PEG-BpAgNPs (4.01 µg/mL) and $IC_{50}$ (4.01 µg/mL + laser light) treatments on A549** | | | |
|---|---|---|---|
| **A549 alone (control)** | **A549 + Laser (L)** | **4.01 µg/mL** | **$IC_{50}$** |
| 4087433 | 3849522 | 7284564 | 73483710 |
| 3917635 | 3939844 | 7440595 | 82398750 |
| 3699190 | 4050140 | 8100133 | 82291750 |
| | | | |
| | | | |

| ROS data following Cum-PEG-BpAgNPs (2.4 µg/mL) and $IC_{50}$ (2.4 µg/mL + laser light) treatments on A549 SC | | | |
|---|---|---|---|
| **A549 SC alone (control)** | **A549 SC + L** | **2.4 µg/mL** | **$IC_{50}$** |
| 4659206 | 5164211 | 5068874 | 95945960 |
| 4449960 | 6430482 | 5267587 | 95538850 |
| 4544285 | 4214530 | 4338956 | 95700020 |

## 3. Annexin V-FTIC and PI cell death analysis data following treatment of A549 and A549 SC $IC_{50}$ doses (Fig.11 C and D)

| Annexin V-FTIC and PI cell death data following Cum-PEG-BpAgNPs (drug) $IC_{50}$ mediated PDT on A549 | | | | | | | | | | | | |
|---|---|---|---|---|---|---|---|---|---|---|---|---|
| | **A549 (untreated/unstained)** | | | **A549 control (untreated/stained)** | | | **A549 + laser (L)** | | | **A549 + $IC_{50}$ (drug + L)** | | |
| **Ql-LL unstained (live cells %)** | 100 | 100 | 100 | 66,4 | 73,1 | 68,7 | 76,5 | 65,5 | 76,5 | 27 | 17,4 | 25,1 |
| **Q2-Early apoptosis %** | 0 | 0 | 0 | 26,3 | 25 | 28,5 | 19,4 | 30,5 | 19,4 | 67,5 | 75 | 67,7 |
| **Q3-Late apoptosis %** | 0 | 0 | 0 | 3,4 | 1,8 | 2,5 | 3,2 | 3,3 | 3,2 | 4,7 | 5,5 | 4 |
| **Q4-necrotic cells %** | 0 | 0 | 0 | 3,9 | 0,1 | 0,2 | 0,9 | 0,7 | 0,9 | 0,7 | 2,1 | 3,2 |
| | | | | | | | | | | | | |
| | | | | | | | | | | | | |
| **Annexin V-FTIC and PI cell death data following Cum-PEG-BpAgNPs (drug) $IC_{50}$ mediated PDT on A549 SC** | | | | | | | | | | | | |
| | **A549 SC (untreated/unstained)** | | | **A549 SC control (untreated/stained)** | | | **A549 SC + laser (L)** | | | **A549 SC + $IC_{50}$ (drug + L)** | | |
| **Ql-LL unstained (live cells %)** | 100 | 100 | 100 | 72,7 | 72,7 | 84,8 | 66,3 | 73,5 | 74 | 37,9 | 36,4 | 37,2 |
| **Q2-Early apoptosis %** | 0 | 0 | 0 | 23,2 | 23,2 | 13,9 | 30,2 | 31,7 | 12,8 | 54,4 | 52,9 | 53,9 |
| **Q3-Late apoptosis %** | 0 | 0 | 0 | 3,4 | 3,4 | 0,9 | 3,2 | 2,5 | 2,4 | 5,3 | 6,9 | 6,9 |
| **Q4-necrotic cells %** | 0 | 0 | 0 | 0,7 | 0,7 | 0,4 | 0,4 | 1,3 | 0,8 | 2,4 | 3,8 | 2 |
| | | | | | | | | | | | | |
